# Significance of PD-L1 in Metastatic Urothelial Carcinoma Treated With Immune Checkpoint Inhibitors: A Systematic Review and Meta-Analysis

**DOI:** 10.1001/jamanetworkopen.2024.1215

**Published:** 2024-03-06

**Authors:** Brigida Anna Maiorano, Massimo Di Maio, Linda Cerbone, Evaristo Maiello, Giuseppe Procopio, Giandomenico Roviello

**Affiliations:** 1 Oncology Unit, IRCCS Casa Sollievo della Sofferenza, San Giovanni Rotondo, Italy; 2 Oncology Department, University of Turin, Turin, Italy; 3 Medical Oncology 1, AOU Città della Salute e della Scienza, Molinette Hospital, Turin, Italy; 4 Oncology Unit, San Camillo Forlanini Hospital, Rome, Italy; 5 Unit of Genito-Urinary Medical Oncology, IRCCS Foundation Istituto Nazionale dei Tumori, Milan, Italy; 6 Department of Health Sciences, Section of Clinical Pharmacology and Oncology, University of Florence, Florence, Italy

## Abstract

**Importance:**

Immune checkpoint inhibitors (ICIs) have broadened the metastatic urothelial carcinoma (mUC) therapeutic scenario. The association of programmed death ligand 1 (PD-L1) with response and survival in patients treated with ICIs is still controversial.

**Objectives:**

To evaluate the association of PD-L1 with response rate and overall survival among patients with mUC treated with ICIs.

**Data Sources:**

PubMed, Embase, American Society of Clinical Oncology and European Society for Medical Oncology Meeting Libraries, and Web of Science were searched up to December 10, 2023.

**Study Selection:**

Two authors independently screened the studies. Included studies were randomized and nonrandomized clinical trials enrolling patients with mUC receiving ICIs with available overall survival (OS), progression-free survival (PFS), or overall response rate (ORR) data, separated between patients with PD-L1–positive and –negative tumors.

**Data Extraction and Synthesis:**

The Preferred Reporting Items for Systematic Reviews and Meta-analyses (PRISMA) reporting guideline was followed. Two reviewers independently extracted data. Fixed- or random-effects models were used depending on the heterogeneity among the studies.

**Main Outcomes and Measures:**

Primary outcomes were odds ratios (ORs) for ORR and hazard ratios (HRs) for OS, comparing patients with PD-L1–positive tumors and patients with PD-L1–negative tumors. Secondary outcomes were the PFS HR between patients with PD-L1–positive and –negative tumors and OS HR between ICI arms and non-ICI arms of only randomized clinical trials.

**Results:**

A total of 14 studies were selected, comprising 5271 patients treated with ICIs (2625 patients had PD-L1–positive tumors). The ORR was 13.8% to 78.6% in patients with PD-L1–positive tumors and 5.1% to 63.2% in patients with PD-L1–negative tumors, with an association between PD-L1 status and ORR favoring patients with PD-L1–positive tumors (OR, 1.94; 95% CI, 1.47-2.56; *P* < .001). Median OS ranged from 8.4 to 24.1 months in patients with PD-L1–positive tumors and from 6.0 to 19.1 months in patients with PD-L1–negative tumors. The pooled HR showed a significant reduction for patients with PD-L1–positive tumors compared with those with PD-L1–negative tumors in the risk of death (HR, 0.71; 95% CI, 0.57-0.89; *P* = .003) and risk of progression (HR, 0.55; 95% CI, 0.44-0.69; *P* < .001) when ICIs were administered. PD-L1 is not likely to be a predictive biomarker of ICI response.

**Conclusions and Relevance:**

This systematic review and meta-analysis suggests that PD-L1 expression is associated with improved ORR, OS, and PFS for patients with mUC who receive ICIs, but it is unlikely to be useful as a predictive biomarker. Developing predictive biomarkers is essential to select patients most likely to benefit from ICIs and avoid toxic effects and financial burden with these agents.

## Key Points

**Question:**

Is programmed death ligand 1 (PD-L1) associated with improved outcomes among patients with urothelial carcinoma treated with immune checkpoint inhibitors (ICIs)?

**Findings:**

In this systematic review and meta-analysis of 14 phase 1 to 3 studies, an association emerged between PD-L1 status and overall response rate, favoring patients with PD-L1–positive tumors. There was a significant reduction in the risk of disease progression and death for patients with PD-L1–positive tumors compared with patients with PD-L1–negative tumors.

**Meaning:**

This study suggests that PD-L1–positive tumors are associataed with improved prognosis among patients with metastatic urothelial carcinoma who recieve ICIs, but PD-L1 is not likely to be a predictive biomarker of ICI response.

## Teaser

This systematic review and meta-analysis evaluates the association of programmed death ligand 1 (PD-L1) with overall response rate and overall survival among patients with metastatic urothelial carcinoma treated with immune checkpoint inhibitors.

## Introduction

Urothelial carcinoma (UC) accounts for almost 600 000 new cases and over 200 000 deaths per year, representing the ninth most common malignant neoplasm worldwide.^1^ Although approximately 70% of UCs are diagnosed at a nonmuscle-invasive stage, most patients develop distant metastases, with poor survival.^2^ In the metastatic setting, platinum-based chemotherapy represents the first-line standard of care. Although several chemotherapy agents have been studied, low response rates (8%-30%) and survival (7-10 months) have been reported after failure of platinum-based chemotherapy.^3^

Immune checkpoint inhibitors (ICIs) were added to the therapeutic armamentarium of metastatic UC (mUC) starting in 2016: 2 programmed cell death 1 (PD1) inhibitors (nivolumab and pembrolizumab) and 3 PD-ligand 1 (PD-L1) inhibitors (atezolizumab, durvalumab, and avelumab) were effective in mUC progressing after platinum-containing chemotherapy. However, in untreated patients, results were not satisfactory compared with platinum-based regimens. More recently, the JAVELIN Bladder 100 study changed the treatment paradigm of mUC after avelumab maintenance was associated with increased survival in patients who responded to platinum-based chemotherapy.^4^

Until now, identifying the best candidates for immunotherapy represents one of the most critical unmet needs in this field. Programmed death ligand 1 (B7-H1 or CD274) is 1 of the 2 ligands of PD1 and a member of the B7 family of type I transmembrane protein receptors.^5^ Historically, it has been the first biomarker associated with better outcomes with ICIs for different solid tumors. The role of PD-L1 expression as a biomarker for identifying patients with mUC who are most likely to benefit from ICIs is still controversial.^6,7^ Therefore, our systematic review and meta-analysis aimed to evaluate the association of PD-L1 with response rate and survival in patients with mUC.

## Methods

### Data Retrieval Strategies

We conducted a systematic review and meta-analysis following the Preferred Reporting Items for Systematic Reviews and Meta-analyses (PRISMA) reporting guideline.^8^ Embase, PubMed, American Society of Clinical Oncology and European Society for Medical Oncology Meeting library databases, and Web of Science were searched. Publications available up to December 10, 2023, were analyzed. The following terms were used: [*urothelial cancer*, *urothelial carcinoma*, *bladder cancer*, *bladder carcinoma*] and [*avelumab*, *durvalumab*, *atezolizumab*, *nivolumab*, *pembrolizumab*]. The search was restricted to the English language (eTable 1 in Supplement 1).

### Population, Intervention, Comparison, and Outcomes

Patients with mUC treated with ICIs were included in our meta-analysis. To test the association of PD-L1 status with prognosis, the experimental group included patients with PD-L1–positive tumors, with criteria for PD-L1 positivity (cutoff and detection methods) established by the individual studies. This group was compared with PD-L1–negative patients. Overall survival (OS) and overall response rate (ORR) were the primary outcomes. Progression-free survival (PFS) was the secondary outcome, alongside OS between ICI arms and non-ICI arms. No correction for multiplicity was applied.

### Inclusion Criteria

Two authors (B.A.M. and G.R.) independently screened the studies. Decisions regarding contentious studies were made in consultation with a third author (M.D.). The inclusion criteria were (1) studies enrolling patients with mUC; (2) use of ICIs as single agents or in combination; (3) availability of OS, PFS, or ORR data; and (4) data of the reported outcomes separated between patients with PD-L1–positive and PD-L1–negative tumors. Studies not reporting the selected outcome for patients with PD-L1–positive and PD-L1–negative tumors, observational studies, animal studies, and studies with a sample size of less than 10 patients were excluded. The analysis was limited to phase 1 to 3 clinical trials (eTable 2 in Supplement 1).

### Data Extraction

Two authors (B.A.M. and G.R.) extracted the relevant data, including trial name and author, publication year, phase, and line of therapy; ICI type and dosage and presence of a control arm; PD-L1 assay and cutoff; total sample size, number of patients with PD-L1–positive and PD-L1–negative tumors, and primary outcomes; and ORR, OS, and PFS in the overall population, patients with PD-L1–positive tumors, and patients with PD-L1–negative tumors.

### Statistical Analysis

Study quality was assessed using the Cochrane tools to assess the bias risk (ROB-2 [Risk of Bias 2] for randomized trials, ROBINS-I [Risk of Bias in Non-randomised Studies—of Interventions] for nonrandomized trials).^9^ The statistical analysis was performed with Revman, version 5.4 (Cochrane Training). The summary estimates were generated using the generic inverse variance and a fixed-effect model (Mantel-Haenszel method) or a random-effect model (DerSimonian-Laird method) depending on the absence or presence of heterogeneity. Statistical heterogeneity was assessed with the *Q* test and the *I*^2^ statistic.^10,11^ *I*^2^ values of 25%, 50%, and 75% were considered for low, moderate, and high heterogeneity, respectively.^12^ When *I*^2^ was less than 40%, the fixed-effects model was used; otherwise, the random-effects model was used. To test the association of PD-L1 status with prognosis, odds ratios (ORs) with 95% CIs for ORR and hazard ratios (HRs) for OS and PFS, comparing patients with PD-L1–positive tumors, and those with PD-L1–negative tumors were calculated for each study. A value of *P* < .05 was considered statistically significant, and all tests were 2-sided. We planned subgroup analyses according to the line of therapy (first, maintenance, second, and beyond), single agent vs combination (anti-PD1 or –PD-L1 plus anticytotoxic T-lymphocyte–associated protein 4 [CTLA4] or chemotherapy or enfortumab vedotin), and according to ICI mechanism of action (anti-PD1 or anti–PD-L1). A sensitivity analysis was performed to assess the stability of the global estimates by moving away 1 study at a time. Moreover, randomized clinical trials were included to assess HR for OS in patients with PD-L1–positive tumors treated with ICIs compared with other treatments in patients with PD-L1–negative tumors.

## Results

### Characteristics of the Included Studies

After the literature search and the inclusion and exclusion criteria screening, a total of 14 studies were selected (eFigure 1 in Supplement 1).^13,14,15,16,17,18,19,20,21,22,23,24,25,26,27,28^ There were 7 phase 1 or 2 trials and 7 randomized phase 3 trials. IMvigor210 consisted of 2 cohorts: cohort 1 included pretreated patients and cohort 2 enrolled untreated patients.^13^ In 5 studies, ICIs were administered to pretreated patients.^14,15,16,17,18,19^ In 8 trials, ICIs were used as first-line treatment and, in 1 case, avelumab was administered as maintenance after at least stability to platinum-based frontline chemotherapy.^20,21,22,23,24,25,26,27^ A total of 8 studies used the anti-PD1 agents nivolumab (n = 3) or pembrolizumab (n = 5).^14,15,16,17,22,23,24,26,27^ In 6 studies, PD-L1 inhibitors were administered (avelumab, 2; atezolizumab, 2; or durvalumab, 2).^13,18,19,20,21,25,26^ In 2 studies, the anti-CTLA4 agents ipilimumab and tremelimumab were added to nivolumab (second line) and durvalumab (first line), respectively; in the first line, atezolizumab, nivolumab, and pembrolizumab were combined with chemotherapy in 3 studies, and pembrolizumab was combined with enfortumab vedotin in 1 study.^17,23,24,25,26,27^

Overall, 5271 patients were treated with ICIs; 2625 had PD-L1 positive tumors, representing 28.5% to 82.0% of the sample. Overall response rate was the primary end point in 5 studies; it was defined as the rate of complete responses and partial responses to treatment.^13,15,16,17,18,20,22^ Overall survival, defined as the time from starting treatment to the patient’s death, was the primary end point in 5 studies.^14,21,23,25,26^ In 5 studies, PFS, defined as the time from randomization to disease progression or patients’ death, whichever occurred first, was explored as a coprimary end point with OS^14,21,23,26,27^ (eTable 3 in Supplement 1). The main characteristics of included studies and efficacy data are listed in the Table.^13,14,15,16,17,18,19,20,21,22,23,24,25,26,27,28^

**Table.** Characteristics of the Included Studies

Source	Trial name	Phase	Sample size			ORR (95% CI), %			mOS (95% CI), mo			PFS (95% CI), mo			PD-L1 cutoff, cell types (detection platform)
			Total, No.	PD-L1 positive, No. (%)	PD-L1 negative, No. (%)	Overall	PD-L1 positive	PD-L1 negative	Overall	PD-L1 positive	PD-L1 negative	Overall	PD-L1 positive	PD-L1 negative	
**Second-line therapy**															
Rosenberg et al,^13^ 2016	IMvigor 210 (cohort 2)	2	310	207 (66.7)	103 (33.3)	14.8 (11-19)	18.0 (13.0-24.0)	8.0 (3.0-15.0)	7.9 (6.6-9.3)	8.8 (7.1-10.6)	NA	2.1 (2.1-2.1)	2.9 (2.1-4.1)	NA	5%, IC (Ventana SP142)
Bellmunt et al,^14^ 2017	KEYNOTE-045	3	260	74 (28.5)	NA	21.1 (16.4-26.5)	21.6 (12.9-32.7)	NA	10.3 (8.0-12.3)	NA	NA	2.1 (2.0-2.2)	NA	NA	CPS ≥10, TC and IC (Dako 22C3)
Sharma et al,^15^ 2017	CheckMate 275	2	265	122 (46)	143 (54)	19.6	23.8 (16.5-32.3)	16.1 (10.5-23.1)	8.6 (6.1-11.3)	11.9 (9.1-19.1)	6.0 (4.4-8.1)	1.9 (1.9-2.3)	3.5 (1.9-3.7)	1.9 (1.7–2.0)	5%, amended to 1%, TC (Dako 28.8)
Sharma et al,^16^ 2016 and Sharma et al,^17^ 2019	CheckMate 032	1/2	78	26 (33.3)	43 (55.1)	N3: 25.6 (16.4-37.8)	26.9 (11.6-47.8)	25.6 (13.5-41.2)	9.9 (7.3-21.1)	12.9 (2.8-NR)	10.4 (6.5-26.0)	2.8 (1.5-5.3)	2.7 (1.2-10.8)	2.8 (1.4-5.9)	1%, TC (Dako 28.8)
			104	31 (29.8)	56 (53.8)	N3+I1: 26.9 (18.7-36.5)	35.5 (19.2-54.6)	25.0 (14.5-38.4)	7.4 (5.6-11.0)	10.8 (4.6-NR)	7.4 (5.0-10.6)	2.6 (1.4-3.9)	3.4 (1.4-11.0)	2.7 (1.4-3.9)	
			92	31 (33.7)	42 (45.7)	N1+I3: 38.0 (28.1-48.8)	58.1 (39.1-75.5)	23.8 (12.1-39.5)	15.3 (10.1-27.6)	24.1 (10.2-NR)	14.9 (5.6-27.6)	4.9 (2.7-6.6)	6.6 (3.8-27.6)	4.3 (1.5-6.4)	
Powles et al,^18^ 2017	STUDY 1108	1/2	191	98 (51.3)	79 (41.4)	17.8 (12.7-24.0)	27.6 (19.0-37.5)	5.1 (1.4-12.5)	18.2 (8.1-NR)	20.0 (11.6-NR)	8.1 (3.1-NR)	1.5 (1.4-1.9)	2.1 (1.4-2.8)	1.4 (1.3-1.5)	25%, TC/IC (Ventana SP263)
Patel et al,^19^ 2018	NCT01772004 (JAVELIN mUC EC)	1b	249	85 (34.1)	135 (54.2)	16.5 (12.1-21.8)	23.8 (15.2-34.3)	12.3 (7.2-19.2)	7.0 (5.9-8.5)	8.4 (6.0-11.3)	6.5 (5.3-10.1)	1.6 (1.4-2.7)	2.2 (1.4-4.1)	1.5 (1.4-2.4)	5%, TC (Dako 73-10)
**First-line therapy**															
Balar et al,^20^ 2017	IMvigor 210 (cohort 1)	2	119	80 (67.0)	39 (33.0)	23.0 (16.0-31.0)	24.0 (15.0-35.0)	21.0 (9.0-36.0)	15.9 (10.4-NR)	12.3 (6.0-NR)	19.1 (9.8-NR)	2.7 (2.1-4.2)	4.1 (2.3-11.8)	2.6 (2.1-5.7)	5%, IC (Ventana SP142)
Galsky et al,^21^ 2020	IMvigor 130	3	451	303 (67.0)	148 (33.0)	Atezolizumab + CT: 47.0 (43.0-52.0)	NA	NA	16 (13.9-18.9)	23.6	14.2	8.2 (6.5-8.3)	8.6	6.5	1% (IC1), 5% (IC2/3) (Ventana SP142)
			352	248 (68.0)	114 (31.0)	Atezolizumab: 23.0 (19.0-28.0)	34.0 (28.0-50.0)	NA	15.7 (13.1-17.8)	NR (17.7-NR)	13.5 (11.1-16.4)	NA	NA	NA	NA
Balar et al,^22^ 2017	KEYNOTE-052	2	317	219 (82.0)	46 (17.0)	28.6 (24.1-33.5)	47.3 (37.7-57.0)	20.3 (15.5-25.8)	11.3 (9.7-13.1)	18.5 (12.2-28.5)	9.7 (7.6-11.5)	2.2 (2.1-3.4)	NA	NA	CPS ≥10%, TC and IC (Dako 22C3)
Powles et al,^23^ 2021	KEYNOTE-361	3	351	160 (52.0)	147 (48.0)	Pembrolizumab + CT: 54.7	NA	NA	17.0 (14.5-19.5)	NA	NA	8.3 (7.5-8.5)	NA	NA	CPS ≥10%, TC and IC (Dako 22C3)
			307	159 (45.0)	192 (55.0)	Pembrolizumab: 30.3	NA	NA	15.6 (12.1-17.9)	16.1 (13.6-19.9)	NA	3.9	NA	NA	NA
Rosenberg et al,^24^ 2020	EV-103	1b/2	45	14 (31.1)	19 (42.2)	73.3	78.6	63.2	NR	NA	NA	12.3	NA	NA	CPS ≥10%, TC and IC (Dako 22C3)
Powles et al,^25^ 2020	DANUBE	3	346	209 (60.0)	137 (40.0)	Durvalumab: 26.9	29.1	23.8	13.2 (10.3-15)	14.4 (10.4-17.3)	10.9 (8.0-14.8)	2.3 (1.9-3.5)	2.4 (1.9-3.7)	1.9	25% TC or 25% IC + 1% TC (Ventana SP263)
			342	205 (60.0)	137 (40.0)	Durvalumab + tremelimumab: 36.6	47.0	21.5	15.1 (13.1-18)	17.9 (14.8-24.2)	11.8 (8.9-15.8)	3.7 (3.4-3.8)	4.1 (3.6-5.7)	2.9	NA
van der Heijden et al,^26^ 2023	CheckMate 901	3	304	111 (36.5)	193 (63.5)	57.6	NA	NA	21.7 (18.6-26.4)	NA	NA	7.9 (7.6-9.5)	NA	NA	1%, TC (Dako 28.8)
Powles et al,^27^ 2023	EV-302/ KEYNOTE-A39	3	438	254 (58.0)	184 (42.0)	67.7 (63.1-72.1)	NA	NA	31.5 (25.4-NR)	NA	NA	12.5 (10.4-16.6)	31.5 (25.4-NR)	NR (22.3-NR)	CPS ≥10, TC and IC (Dako 22C3)
**First-line maintenance**															
Powles et al,^28^ 2020	JAVELIN Bladder 100	3	350	189 (54.0)	139 (40.0)	9.7 (6.8-13.3)	13.8 (9.2-19.5)	NA	21.4 (18.9-26.1)	NR (20.3-NR)	18.8 (13.3-22.5)	3.7 (3.5-5.5)	5.7 (3.7-7.4)	3.0 (2.0-3.7)	25%, TC/IC (Ventana SP263)

Abbreviations: CPS, combined positive score; CT, chemotherapy; IC, immune cells; mOS, median overall survival; NA, not available; NR, not reached; N1+I3, nivolumab 1 mg/kg + ipilimumab 3 mg/kg; N3, nivolumab 3 mg/kg; N3+I1, nivolumab 3 mg/kg + ipilimumab 1 mg/kg; ORR, overall response rate; PD-L1, programmed death ligand 1; PFS, progression-free survival; TC, tumor cells.

### ORR of Patients With PD-L1–Positive vs PD-L1–Negative Tumors

Data for ORR in patients with PD-L1–positive vs PD-L1–negative tumors were available in 10 studies.^13,14,15,16,17,18,19,20,22,24,25,26^ Among the 3068 treated patients, 1590 had PD-L1 positive tumors and 1125 had PD-L1 negative tumors; information regarding PD-L1 status was unavailable in 353 cases. The ORR ranged from 13.8% to 78.6% in patients with PD-L1–positive tumors and from 5.1% to 63.2% in patients with PD-L1–negative tumors (Table).^13,14,15,16,17,18,19,20,21,22,23,24,25,26,27,28^

**Figure 1.**
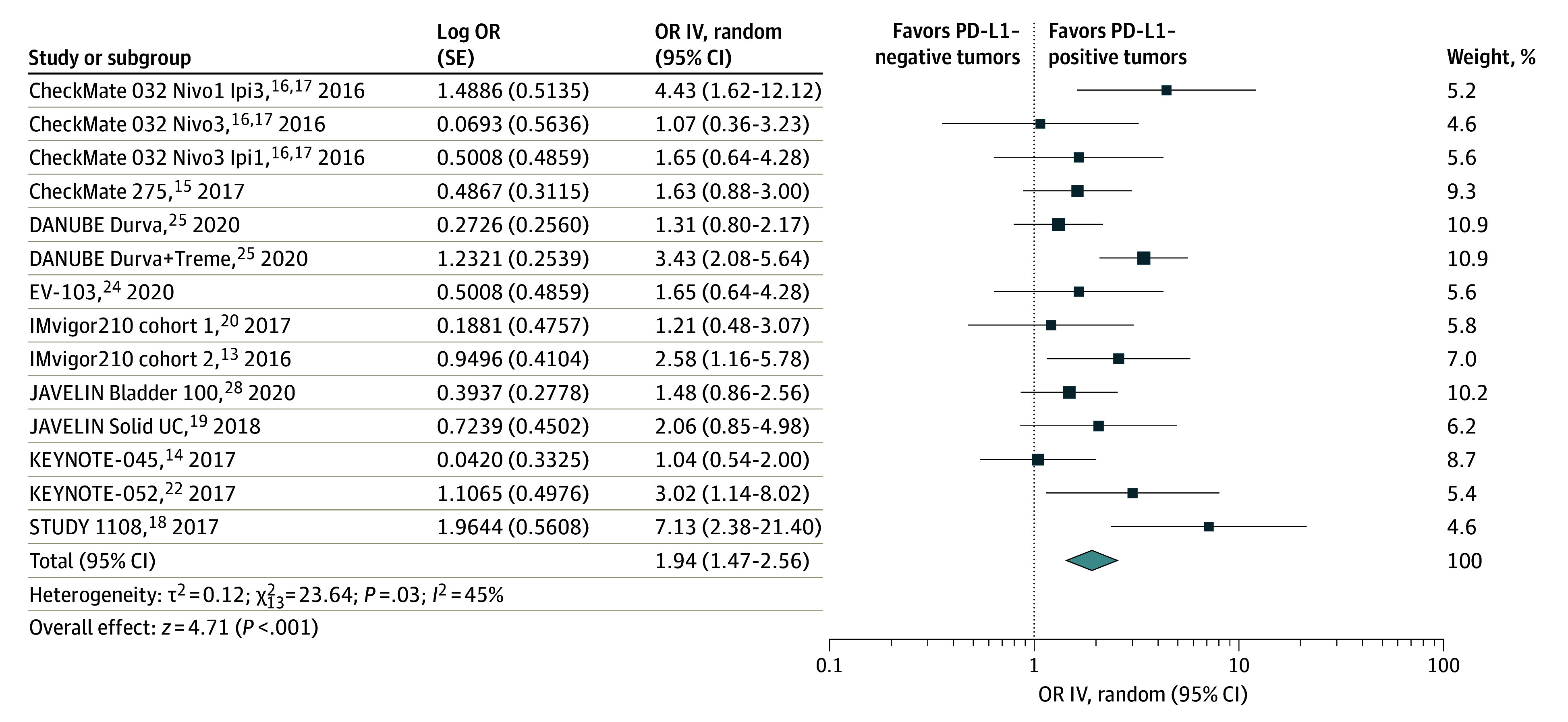
Overall Response Rate for Patients With Programmed Death Ligand 1 (PD-L1)–Positive vs PD-L1–Negative Tumors The diamond indicates the pooled estimate, derived from the generic inverse variance (IV) and a random-effects model. Durva indicates durvalumab; Ipi, ipilimumab; Nivo, nivolumab; OR, odds ratio; and Treme, tremelimumab.

We found an association between PD-L1 status and ORR favoring patients with PD-L1–positive tumors (OR, 1.94, 95% CI, 1.47-2.56; *P* < .001; random-effects) (Figure 1). Low heterogeneity was observed among the studies (*I^2^* = 45%; *P* = .03).

### OS of Patients With PD-L1–Positive vs PD-L1–Negative Tumors

Data for OS were available from 12 studies and 4909 patients, of whom 2267 had PD-L1–positive and 1888 had PD-L1–negative tumors.^13,14,15,16,17,18,19,20,21,23,25,26,27,28^ Among patients with PD-L1–positive tumors, median OS ranged from 8.4 to 24.1 months. Among patients with PD-L1–negative tumors, median OS ranged from 6.0 to 19.1 months (Table).^13,14,15,16,17,18,19,20,21,22,23,24,25,26,27,28^

**Figure 2.**
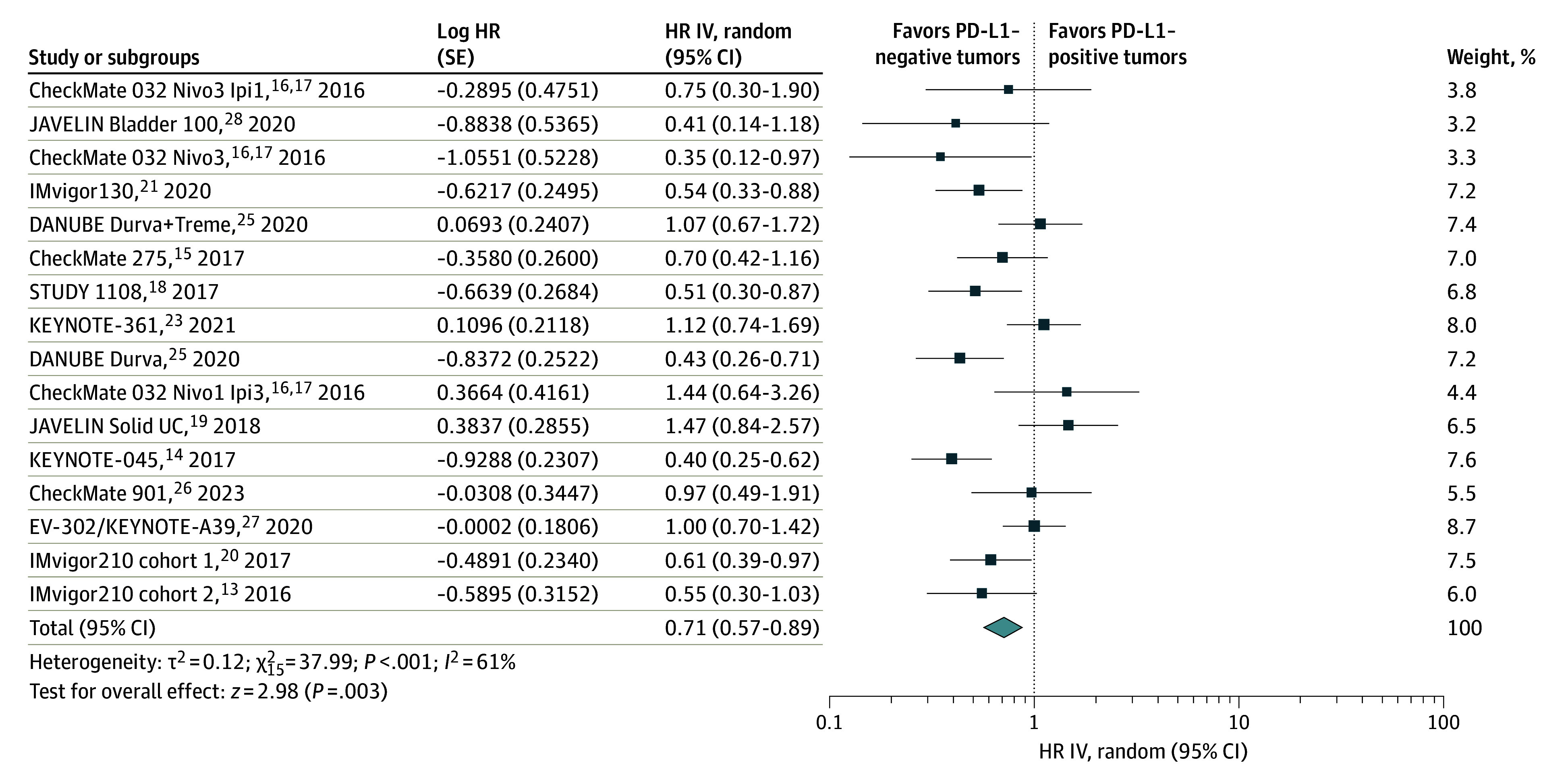
Overall Survival of Patients With Programmed Death Ligand 1 (PD-L1)–Positive vs PD-L1–Negative Tumors The diamond indicates the pooled estimate, derived from the generic inverse variance (IV) and a random-effects model. Durva indicates durvalumab; HR, hazard ratio; Ipi, ipilimumab; Nivo, nivolumab; and Treme, tremelimumab.

The pooled HR showed a significant reduction in the risk of death for patients with PD-L1–positive tumors compared with those with PD-L1–negative tumors (HR, 0.71; 95% CI, 0.57-0.89; *P* = .003; random-effects) (Figure 2). There was a moderate heterogeneity among the studies (*I*^2^ = 61%; *P* < .001).

### PFS of Patients With PD-L1–Positive vs PD-L1–Negative Tumors

**Figure 3.**
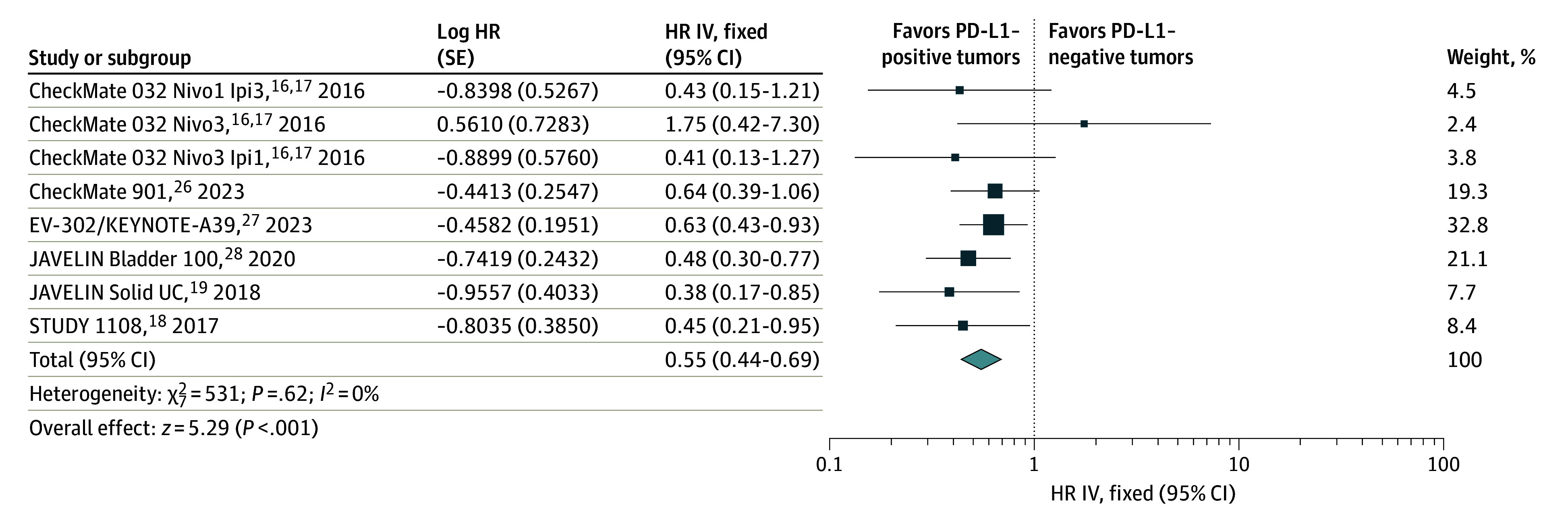
Progression-Free Survival of Patients With Programmed Death Ligand 1 (PD-L1)–Positive vs PD-L1–Negative Tumors The diamond indicates the pooled estimate, derived from the generic inverse variance (IV) and a fixed-effects model. HR indicates hazard ratio; Ipi, ipilimumab; and Nivo, nivolumab.

Six studies reported PFS for a total of 1638 patients (884 with PD-L1 positive tumors and 754 with PD-L1 negative tumors).^16,17,18,19,26,27,28^ Compared with patients with PD-L1–negative tumors, those with PD-L1–positive tumors had improved PFS (HR, 0.55; 95% CI, 0.44-0.69; *P* < .001; fixed-effects) (Figure 3). No heterogeneity was observed (*I*^2^ = 0%; *P* = .62).

### Subgroup Analyses

We performed subgroup analyses for ORR and OS to test the source of heterogeneity, considering ICIs’ mechanisms of action, lines of therapy, and single agents vs combination (eTables 4 and 5 in Supplement 1). No differences were found in terms of ICIs’ mechanisms of action (ORR for anti-PD1 vs anti–PD-L1: OR, 1.83 [95% CI, 1.39-2.40]; *P* = .69; OS for anti-PD1 vs anti–PD-L1: OR, 0.68 [95% CI, 0.55-0.84]; *P* = .92), lines of therapy (ORR for first vs second vs maintenance: OR, 1.83 [95% CI, 1.39-2.40]; *P* = .73; OS for first vs second vs maintenance: 0.68 [95% CI, 0.55-0.84]; *P* = .07 for OS), or single agents vs combinations (ORR: OR, 1.94 [95% CI, 1.47-2.56]; *P* = .08; OS: OR, 0.69 [95% CI, 0.55-0.86]; *P* = .29).

### Association of PD-L1 With OS Between ICIs and Non-ICIs in mUC

**Figure 4.**
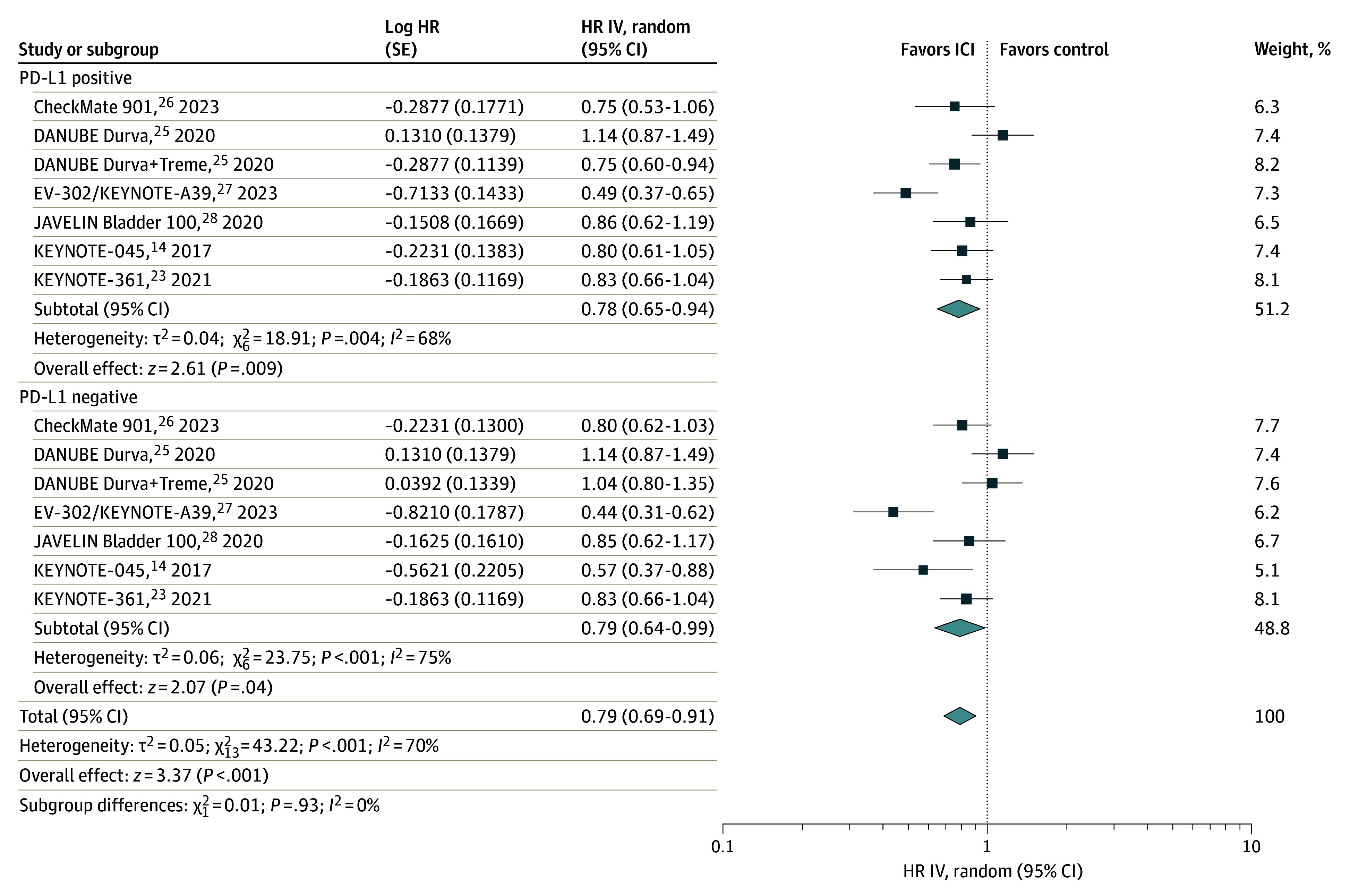
Overall Survival of Patients With Metastatic Urothelial Carcinoma Receiving Immune Checkpoint Inhibitors (ICIs) vs Non-ICIs by Programmed Death Ligand 1 (PD-L1) Status The diamonds indicate the pooled estimates, derived from the generic inverse variance (IV) and a random-effects model. Durva indicates durvalumab; HR, hazard ratio; and Treme, tremelimumab.

We subsequently aimed to analyze the association of PD-L1 with OS between ICIs and non-ICIs from randomized clinical trials. Six studies were selected comparing ICIs with different treatments (chemotherapy or best supportive care) in the control arm. No differences emerged in the PD-L1–positive compared with the PD-L1–negative subgroup (HR, 0.79 [95% CI, 0.69-0.91]; *P* = .93) for survival with ICIs compared with other treatments (Figure 4).

### Risk of Bias of the Included Studies

Globally, the quality of the studies was high. Among the nonrandomized studies, the principal bias depended on missing data, whereas performance biases were most frequently detected in randomized clinical trials (eFigure 2A and B in Supplement 1).

Overall, there was a low asymmetry in the distribution of the published studies (eFigure 3A and B in Supplement 1). We performed a sensitivity analysis to test the association of the single studies with the overall results. The global estimates were not changed after removing every single study at a time (eFigure 4A-C in Supplement 1).

## Discussion

The results of our systematic review and meta-analysis demonstrate a positive association of PD-L1 with ORR, OS, and PFS in patients with mUC treated with ICIs. However, our results suggest that PD-L1 is not a valid predictive biomarker for patients’ selection in this tumor type.

Urothelial carcinoma accounts for approximately 3% of tumors worldwide. For over 2 decades, platinum-based chemotherapy represented the standard of care in first-line therapy.^29,30^ Failure to respond to platinum-based first-line chemotherapy often implies a significant physical dysfunction and quality of life impairment. As a result, only 1 of 3 patients reaches further lines of treatment, with single agents ensuring a median survival of approximately 6 months and ORR of less than 10%.^31,32^ The introduction of ICIs heralds a new era for mUC treatment.^4,33^ First, pembrolizumab improved survival over chemotherapy in pretreated patients.^4,13,14,15,16,17,18,19,33^ Subsequently, in the US, pembrolizumab was authorized as first-line treatment for patients with mUC and PD-L1–positive tumors who were ineligible for cisplatin or for any patients with mUC who were ineligible for platinum, regardless of PD-L1 expression.^4,20,21,22,23,33,34^ In 2020, the US Food and Drug Administration (FDA) granted breakthrough approval to the first-line combination of pembrolizumab and the antibody-drug conjugate enfortumab vedotin for patients who were ineligible for cisplatin, and avelumab was approved by the FDA and European Medical Agency as maintenance treatment after reaching at least disease stability with platinum-based chemotherapy.^4,24,26^

Developing biomarkers with a prognostic role for the outcomes of patients with mUC is still an unmet need. This issue is particularly relevant if we address the costs of such agents. Programmed death ligand 1 expression has been reported in 20% to 30% of patients with UC, often associated with higher disease stages.^35^ Moreover, PD-L1 positivity, especially at higher levels of expression, is negatively prognostic for OS and disease-free survival in UC, particularly bladder cancer.^36,37^ In other reports, high PD-L1 expression on immune cells has been linked to a more favorable prognosis.^37^ Our systematic review and meta-analysis results for ORR, OS, and PFS indicate a positive prognostic role of PD-L1 when ICIs are administered. This correlation seems independent of the type of ICI, the use of ICIs as single agents or in association with other drugs, and the treatment line (eTables 4 and 5 in Supplement 1).

However, approximately 1 in 5 PD-L1–negative patients were also responsive to ICIs. We cannot ignore these data, especially considering response rates and survival that are far longer than with chemotherapeutic agents in second-line or maintenance settings (Table).^13,14,15,16,17,18,19,20,21,22,23,24,25,26,27,28^ Therefore, despite the association with prognosis, it seems unlikely to propose PD-L1 as the only marker to guide the use of ICIs in mUC. However, novel biomarkers such as tumor mutational burden, The Cancer Genome Atlas groups, and genetic and immunologic classifications should be considered. In this regard, fibroblast growth factor receptor (FGFR)–targeting agents were purposed to be potentially synergistic with ICIs, as the FGFR pathway interacts with innate and adaptive immunity.^38^ Initially, trials have investigated sequential FGFR inhibition after progression with ICI treatment, demonstrating higher responses associated with UC tumor microenvironment modifications, especially on the lymphocyte side.^39,40^ A good prognostic role has been associated with FGFR, prevalent in the luminal-papillary UC subtype with a less general aggressiveness but a worse response to chemotherapy and lower PD-L1 levels.^38,40^ Over the prognostic role, such targets offer additive efficacy combined with ICIs; a valid example is the antibody-drug conjugate enfortumab vedotin targeting Nectin-4—a tumor-associated antigen expressed by 97% of UC—that showed an ORR of more than 73% in combination with pembrolizumab in the EV-103 trial, receiving the FDA breakthrough therapy designation for cisplatin-ineligible naive patients with mUC, and is a practice-changing candidate after the EV-302/KEYNOTE-A39 results.^28,41,42^

### Limitations

Our meta-analysis has some limitations. A significant limitation is the heterogeneity between the studies. First and foremost, the studies assayed PD-L1 using tumor cells, immune cells, or both to assess PD-L1, and even the cutoff for defining PD-L1 positivity differed broadly. Four PD-L1 assays have been used in mUC clinical trials (Table).^13,14,15,16,17,18,19,20,21,22,23,24,25,26,27,28^ STUDY 1108 with durvalumab used the Ventana SP263 system, considering PD-L1 positivity on tumor or immune cells (cutoff, 25%), with a difference for ORR between patients with PD-L1–negative tumors that reached only 5.1% and a difference for ORR between patients with PD-L1–positive tumors of 27.8%; similarly, OS ranged from 8.1 to 20.0 months between patients with PD-L1–negative tumors and those with PD-L1–positive tumors.^18^ The association was even more relevant in first-line treatment, with an ORR of 47% for durvalumab plus tremelimumab in patients with PD-L1–positive tumors in the DANUBE trial.^25^ With the same test in the maintenance setting, a more significant effect was observed in patients with PD-L1–positive tumors (median OS not reached), and survival was improved compared with best supportive care in PD-L1–negative patients (18.8 months).^26^ IMvigor210 and IMvigor130 used the Ventana SP142 platform, evaluating the qualitative immunohistochemical expression of PD-L1 on tumor-infiltrating immune cells, with a cutoff of 5% in IMvigor210 and 1% in Imvigor130. Whereas PD-L1 positivity correlated with a higher ORR in second-line treatment, similar percentages were detected in first-line treatment between patients with PD-L1–positive and PD-L1–negative tumors.^13,20,21^ In the CheckMate studies, assessing PD-L1 through the Dako 28.8 system considering expression on tumor cells, the ORR and OS improvement was more relevant in patients with PD-L1–positive tumors with nivolumab combination treatments.^15,16,17,27^ Responses were almost doubled in patients with PD-L1–positive tumors compared with those with PD-L1–negative tumors after ICIs when Dako platforms (22C3, with combined proportion score >10% on tumor and immune cells; 73-10, cutoff 5% on tumor cells) were considered, but the effect on OS was not so relevant with second-line avelumab compared with first-line pembrolizumab.^19,22,23^ The efficacy results were outperforming for the first-line combination of pembrolizumab and enfortumab vedotin, independent from PD-L1 status.^28^

The lack of standardization and variability of expression for PD-L1 among tumor samples may have limited our analysis. However, we did not split studies according to the PD-L1 assay, as it has already been reported that a good correlation exists between the platforms and detection methods.^43,44,45^ This is useful even in the case of multiple test use between laboratories. Another possible limitation could be the time from archival tissue used to detect PD-L1 and the ICI administration. We included both treatment-naive and pretreated patients receiving chemotherapy in the metastatic or perioperative setting. It has already been evidenced that chemotherapy could alter PD-L1 expression.^46^ Moreover, different samples were used for PD-L1 analysis in the selected trials. For example, the CheckMate 032 trial allowed fresh or archived specimens within 3 months from starting nivolumab treatment, whereas KEYNOTE-045 did not apply restrictions on the samples’ age.^14,16,17^ A further limitation could be the different PD-L1 expression between primary and metastatic sites or the intratumoral heterogeneity of PD-L1, influenced by factors such as the tumor microenvironment.^47^ Besides the surface expression, other variables, such as tumor dimension or posttranslational modifications of PD-L1, such as the *N*-glycosylation, could influence receptor detectability.^48^ The use of more accurate techniques, such as the evaluation of circulating tumor cells, could help overcome the limitations of the actual PD-L1 testing and allow real-time and longitudinal monitoring of this biomarker.

Another significant limitation is the exclusion of other studies performed in this setting, as data were not grouped for PD-L1 status (eFigure 1 in Supplement 1). Furthermore, most studies were not randomized, lowering the quality of our evidence, and the meta-analysis was carried out with aggregate rather than individual patients’ data. Finally, further data will come from studies in the adjuvant setting; in the CheckMate 274, PD-L1 confirms a prognostic role for disease-free survival both in the intention-to-treat population and in patients with PD-L1–positive tumors (disease-free survival at 6 months: 74.9% in the intention-to-treat population; HR, 0.70 [95% CI, 0.55-0.90]; 74.5% in patients with PD-L1–positive tumors; HR, 0.55 [95% CI, 0.35-0.85]).^49^

## Conclusions

Our systematic review and meta-analysis demonstrates that PD-L1 expression is associated with improved ORR, OS, and PFS in patients with mUC who receive ICIs targeting PD1 and PD-L1. Results are significant for ICIs used both as first-line and second-line treatment. No predictive role of PD-L1 is indicated by our results. The development of predictive biomarkers is of utmost importance to select patients most likely to benefit from ICIs, avoiding toxic effects and financial burden with this type of treatment and justifying routine biomarkers analysis in the clinical practice.

## Notes

Supplement 1. **eTable 1.** Search Strings for the Used Electronic Databases **eTable 2.** PICOS Structure for Study Selection **eFigure 1.** PRISMA Flowchart of the Selection Process **eTable 3.** Baseline Characteristics of the Included Studies **eTable 4.** Subgroup Analyses for ORR **eTable 5.** Subgroup Analyses for OS **eFigure 2.** Risk of Bias Tools for (A) Not-Randomized (ROBINS-I) and (B) Randomized Studies (ROB-2) **eFigure 3.** Funnel Plot of Selected Studies for (A) ORR, (B) OS, (C) PFS **eFigure 4.** Sensitivity Analysis for (A) ORR, (B) OS, and (C) PFS by Removing One Study at a Time

Supplement 2.
Nonauthor Collaborators

Supplement 3.
Data Sharing Statement
