# Supplementary material for: Significance of PD-L1 in Metastatic Urothelial Carcinoma Treated With Immune Checkpoint Inhibitors: A Systematic Review and Meta-Analysis
Source: JAMA Netw Open. 2024 Mar 6;7(3):e241215. doi: 10.1001/jamanetworkopen.2024.1215 (PMC10918499; doi:10.1001/jamanetworkopen.2024.1215)
Supplement: Supplement 1. — eTable 1. Search Strings for the Used Electronic Databases eTable 2. PICOS Structure for Study Selection eFigure 1. PRISMA Flowchart of the Selection Process eTable 3. Baseline Characteristics of the Included Studies eTable 4. Subgroup Analyses for ORR eTable 5. Subgroup Analyses for OS eFigure 2. Risk of Bias Tools for (A) Not-Randomized (ROBINS-I) and (B) Randomized Studies (ROB-2) eFigure 3. Funnel Plot of Selected Studies for (A) ORR, (B) OS, (C) PFS eFigure 4. Sensitivity Analysis for (A) ORR, (B) OS, and (C) PFS by Removing One Study at a Time [file jamanetwopen-e241215-s001.pdf]

Supplementary Online Content

Maiorano BA, Di Maio M, Cerbone L, Maiello E, Procopio G, Roviello G; MeetURO Group. Significance of PD-L1 in metastatic urothelial carcinoma treated with immune checkpoint inhibitors: a systematic review and meta-analysis. *JAMA Netw Open.* 2024;7(3):e241215.
doi:10.1001/jamanetworkopen.2024.1215

eTable 1. Search Strings for the Used Electronic Databases
eTable 2. PICOS Structure for Study Selection
eFigure 1. PRISMA Flowchart of the Selection Process
eTable 3. Baseline Characteristics of the Included Studies
eTable 4. Subgroup Analyses for ORR
eTable 5. Subgroup Analyses for OS
eFigure 2. Risk of Bias Tools for (A) Not-Randomized (ROBINS-I) and (B) Randomized Studies (ROB-2)
eFigure 3. Funnel Plot of Selected Studies for (A) ORR, (B) OS, (C) PFS
eFigure 4. Sensitivity Analysis for (A) ORR, (B) OS, and (C) PFS by Removing One Study at a Time

This supplementary material has been provided by the authors to give readers additional information about their work.

© 2024 Maiorano BA et al. *JAMA Network Open.*

eTable 1. Search Strings for the Used Electronic Databases

**Electronic databases**	**Search string**
Pubmed	("Immune checkpoint inhibitor" OR “Immune checkpoint inhibitors” OR ICI OR avelumab OR durvalumab OR atezolizumab OR nivolumab OR pembrolizumab) AND ("urothelial cancer" OR "urothelial carcinoma" OR uroth* OR “bladder cancer” OR “bladder carcinoma” OR bladd*)<br>Filters: English
Web of Science	ALL=(((Immune checkpoint inhibitor) OR (Immune checkpoint inhibitors) OR (ICI) (avelumab) OR (durvalumab) OR (atezolizumab) OR (nivolumab) OR (pembrolizumab)) AND ((urothelial cancer) OR (urothelial carcinoma) OR (bladder cancer) OR (bladder carcinoma))
Scopus	((“Immune checkpoint inhibitor” OR “Immune checkpoint inhibitors” OR “ICI” OR “avelumab” OR “durvalumab” OR “atezolizumab” OR “nivolumab” OR “pembrolizumab”) AND (“urothelial cancer” OR “urothelial carcinoma” OR “bladder cancer” OR “bladder carcinoma”))<br>AND (LIMIT-TO (LANGUAGE , "English"))

© 2024 Maiorano BA et al. *JAMA Network Open.*

eTable 2. PICOS Structure for Study Selection

**P**atients	Patients metastatic urothelial carcinoma treated with immune checkpoint inhibitors (ICIs)
**I**ntervention	ICIs in PD-L1 positive patients
**C**omparison	PD-L1 negative patients
**O**utcome(s)	Overall survival (OS), overall response rate (ORR), progression-free survival (PFS)
**S**tudy Design	Phase I-III clinical trials

© 2024 Maiorano BA et al. *JAMA Network Open.*

eFigure 1. PRISMA Flowchart of the Selection Process

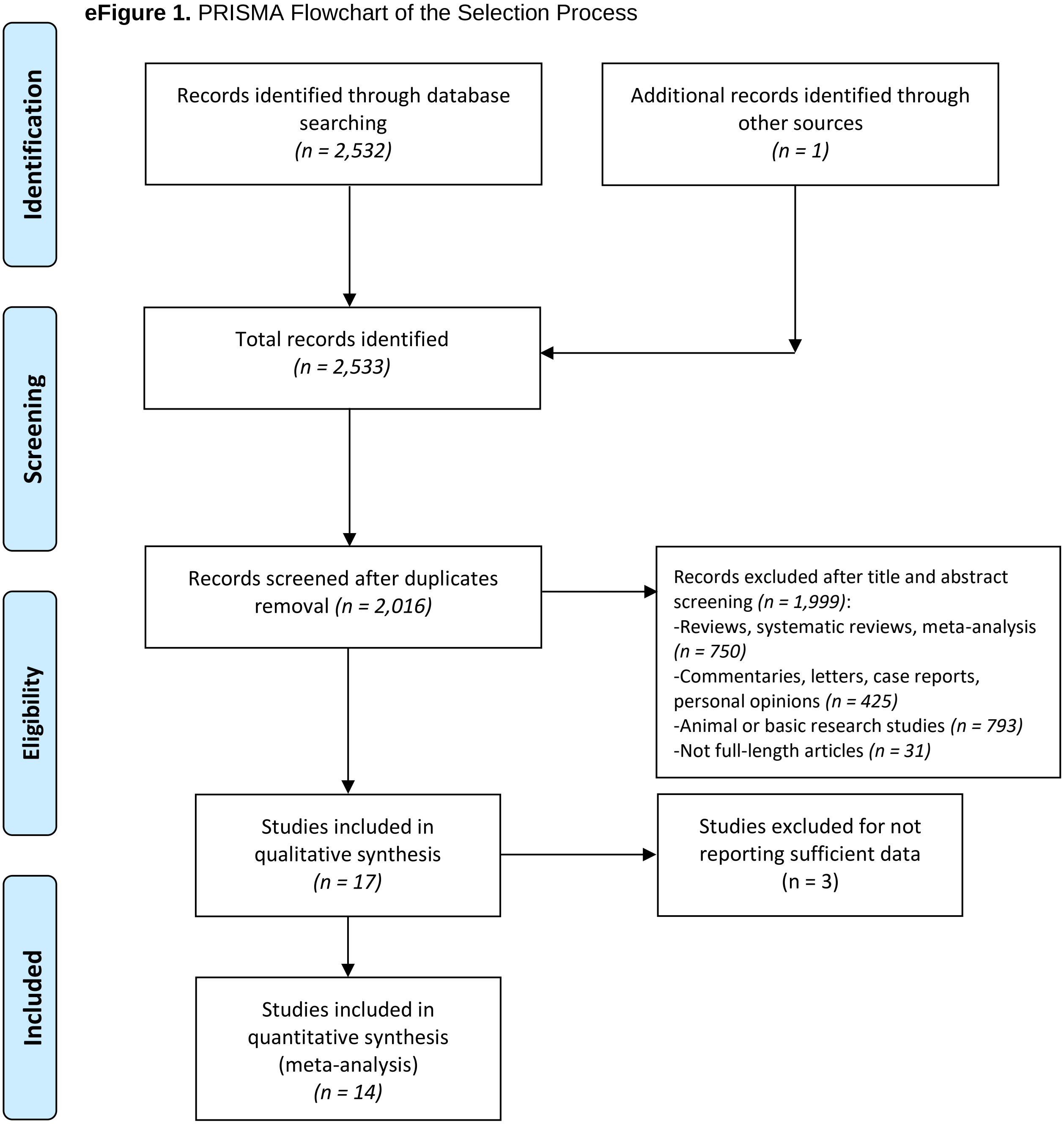

© 2024 Maiorano BA et al. *JAMA Network Open.*

eTable 3. Baseline Characteristics of the Included Studies

Author's name - year	Trial	Line	Primary endpoint	ICI Therapy	Dosage	Control Group
Rosenberg 2016 [13]	IMvigor 210 (Cohort 2)	2	ORR	Atezolizumab	1200 mg q3w	None
Bellmunt 2017 [14]	KEYNOTE-045	2	OS, PFS (overall, PD-L1$^+$)	Pembrolizumab	200 mg q3w	CT
Sharma 2017 [15]	CheckMate 275	2	ORR (overall, PD-L1$^+$)	Nivolumab	3 mg/kg q2w	None
Sharma 2016 [16,17]	CheckMate 032	2	ORR	Nivolumab (NIVO3)	3 mg/kg q2w	None
				Nivolumab + Ipilimumab (NIVO3+IPI1)	Nivo 3 mg/kg, Ipi 1 mg/kg	
				Nivolumab + Ipilimumab (NIVO1+IPI3)	Nivo 1 mg/kg, Ipi 3 mg/kg	
Powles 2017 [18]	STUDY 1108	2	Safety, ORR	Durvalumab	10 mg/kg q2w	None
Patel 2018 [19]	JAVELIN Solid Tumor (mUC EC)	2	Safety	Avelumab	10 mg/kg q2w	None
Balar 2017 [20]	IMvigor 210 (cohort 1)	1 (cis-unfit)	ORR	Atezolizumab	1200 mg q3w	None
Galsky 2020 [21]	IMvigor 130	1	PFS, OS (Atezo +CT vs. CT) → OS (Atezo vs. CT)	Atezolizumab + CT	Atezo 1200 mg q3w	CT + PBO
				Atezolizumab		
Balar 2017 [22]	KEYNOTE-052	1 (cis-unfit)	ORR	Pembrolizumab	200 mg q3w	None
Powles 2021 [23]	KEYNOTE-361	1	OS, PFS	Pembrolizumab	Pembro 200 mg q3w	CT
				Pembrolizumab + CT		
Rosenberg 2020 [24]	EV-103	1 (cis-unfit)	Safety	Pembrolizumab + Enfortumab vedotin	Pembro 200 mg q3w	None
Powles 2020 [25]	DANUBE	1	OS PD-L1+ (Durva vs. CT), OS overall (Durva + Treme vs. CT)	Durvalumab	Durva 1500 mg q4w, treme 75 mg q4w	CT
				Durvalumab + Tremelimumab		
van der Heijden 2023 [26]	CheckMate 901	1	OS, PFS	Nivolumab + CT	Nivo 360 mg q3w x 6 → Nivo 480 mg q4w	CT
Powles 2023 [27]	EV-302/ KEYNOTE-A39	1	OS, PFS	Pembrolizumab + enfortumab vedotin	Pembro 200 mg q3w	CT
Powles 2020 [28]	JAVELIN Bladder 100	1M	OS (overall, PD-L1$^+$)	Avelumab	10 mg/kg q2w	BSC

BSC: best supportive care; CT: chemotherapy; ICI: immune checkpoint inhibitor; ORR: overall response rate; OS: overall survival; PD-L1: programmed cell death-ligand 1; PFS: progression-free survival q2/3/4w: every 2/3/4 weeks; 1M: first-line maintenance

© 2024 Maiorano BA et al. *JAMA Network Open.*

eTable 4. Subgroup Analyses for ORR

Subgroups	ORR, OR (95% CI)	Total	Subgroup differences
ICIs mechanism of action			
Anti-PD1	1.71 (1.19-2.46)	1.83 (1.39-2.4)	*P=0.69*
Anti-PD-L1	1.91 (1.28-2.87)		
Line of therapy			
First line	1.97 (1.21-3.21)	1.83 (1.39-2.4)	*P=0.73*
Second line	1.83 (1.23-2.72)		
Maintenance	1.48 (0.86-2.56)		
Single ICIs vs combo			
ICI single agent	1.70 (1.27-2.27)	1.94 (1.47-2.56)	*P=0.08*
ICI combinations	2.74 (1.75-4.27)		

CI: confidence interval; ICI: immune checkpoint inhibitor; ORR; overall response rate; PD-(L)1: programmed cell death (ligand) 1

© 2024 Maiorano BA et al. *JAMA Network Open.*

eTable 5. Subgroup Analyses for OS

Subgroups	OS, HR (95% CI)	Total	Subgroup differences
ICIs mechanism of action			
Anti-PD1	0.70 (0.55-0.90)	0.68 (0.55-0.84)	*P=0.92*
Anti-PD-L1	0.69 (0.48-0.98)		
Line of therapy			
First line	0.68 (0.52-0.88)	0.68 (0.55-0.84)	*P=0.07*
Second line	0.75 (0.54-1.03)		
Maintenance	0.40 (0.25-0.62)		
Single ICIs vs combo			
ICI single agent	0.77 (0.55-1.10)	0.69 (0.55-0.86)	*P=0.29*
ICI combinations	0.61 (0.48-0.79)		

CI: confidence interval; ICI: immune checkpoint inhibitor; OS; overall survival; PD-(L)1: programmed cell death (ligand) 1

© 2024 Maiorano BA et al. *JAMA Network Open.*

A

	Confounding bias	Selection bias	Classification intervention bias	Deviation from intended intervention	Missing data bias	Measure outcome bias	Selected outcome bias	Overall bias
IMvigor 210	+	+	+	+	+	?	+	+
CheckMate 275	+	+	?	+	+	+	+	+
STUDY 1108	+	?	+	+	+	+	+	+
JAVELIN solid tumor (mUC EC)	+	+	+	+	+	+	+	+
KEYNOTE-052	+	+	+	+	?	+	+	+
EV-103	+	+	+	+	?	+	?	?

B

	Selection bias	Allocation bias	Performance bias	Detection bias	Attrition bias	Reporting bias	Other bias
KEYNOTE-045	+	?	?	+	+	+	+
IMvigor130	+	+	+	+	+	+	+
CheckMate 032	+	?	?	?	+	+	+
KEYNOTE-361	+	+	?	+	+	+	+
DANUBE	+	+	+	?	+	+	+
JAVELIN BLADDER 100	+	?	+	+	+	+	+

eFigure 2. Risk of Bias Tools for (A) Not-Randomized (ROBINS-I) and (B) Randomized Studies (ROB-2)

© 2024 Maiorano BA et al. *JAMA Network Open.*

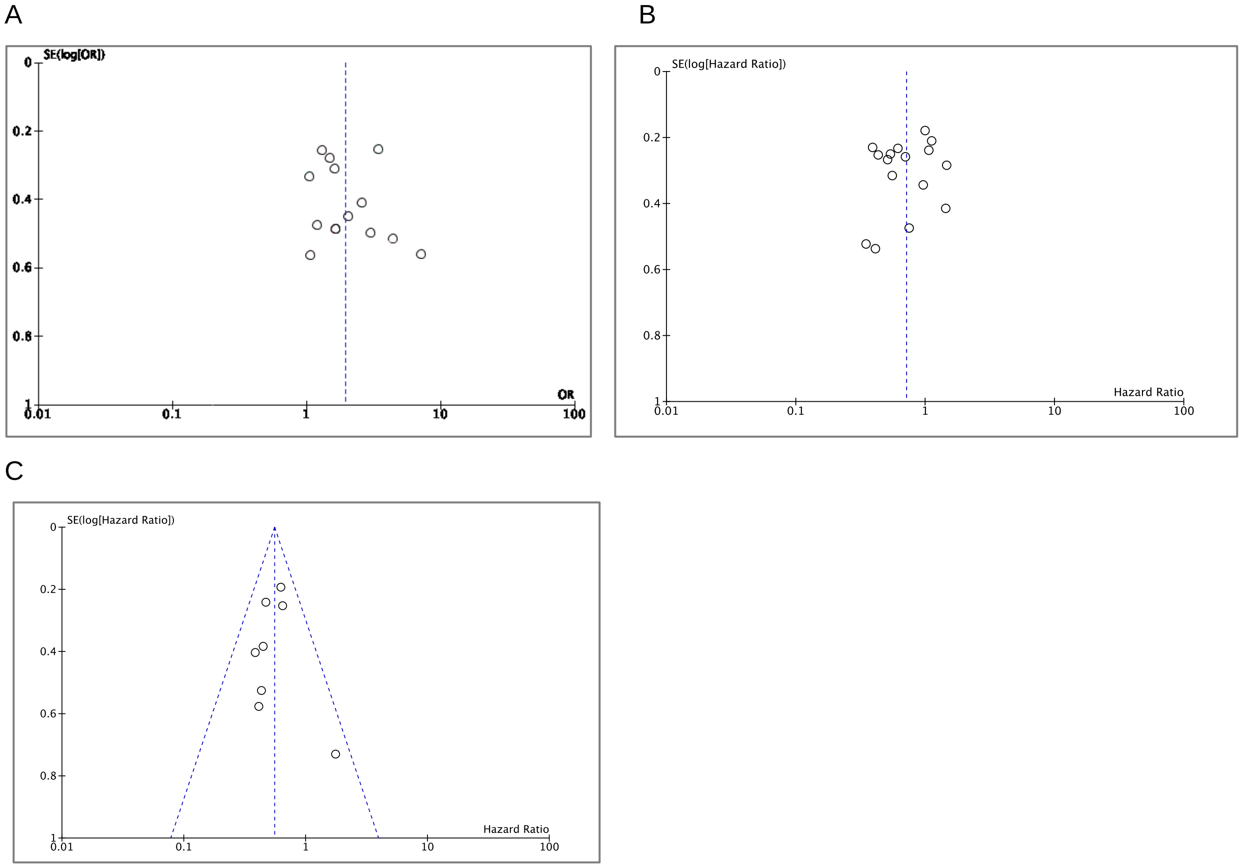

eFigure 3. Funnel Plot of Selected Studies for (A) ORR, (B) OS, (C) PFS

© 2024 Maiorano BA et al. *JAMA Network Open.*

A

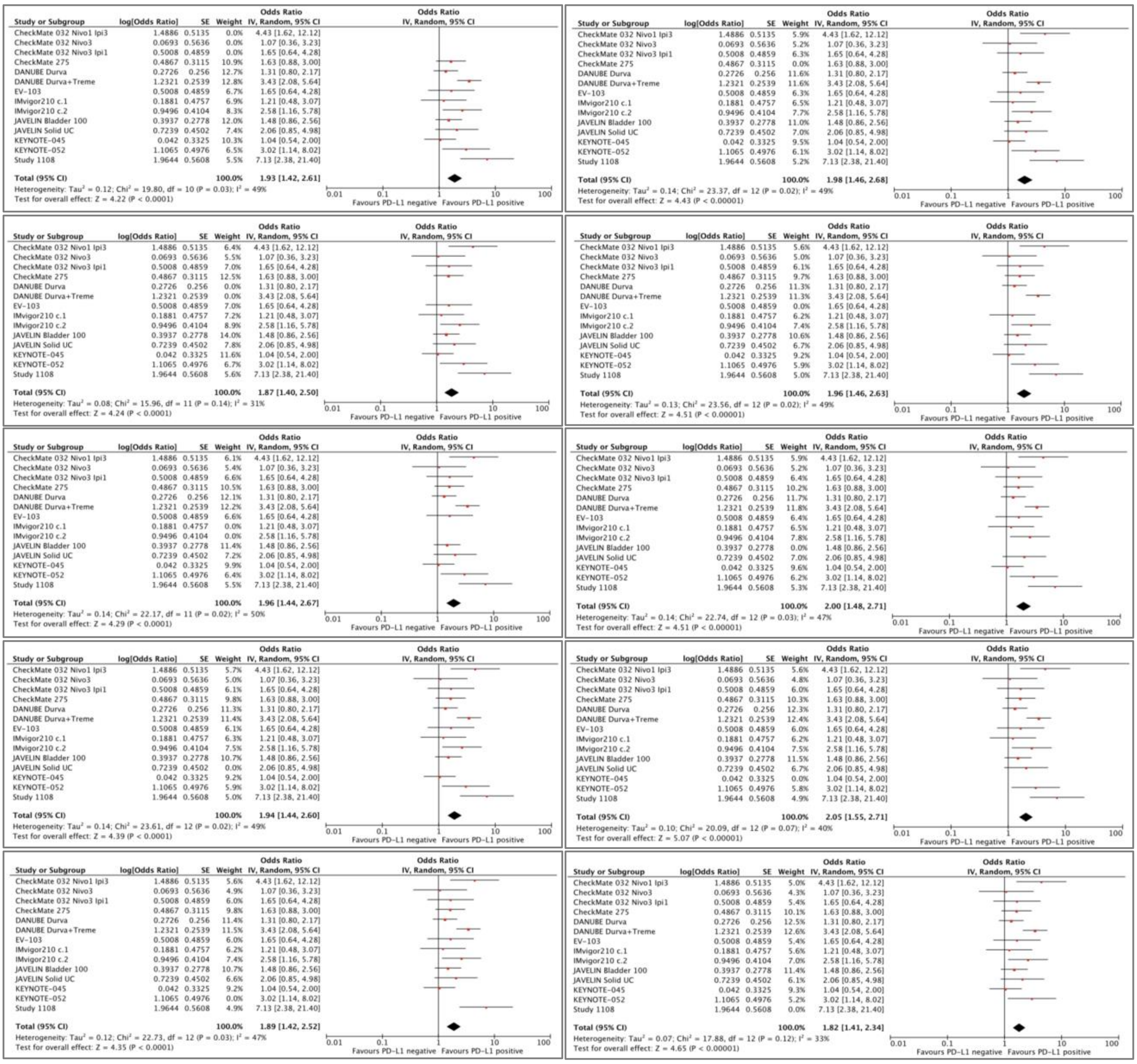

B

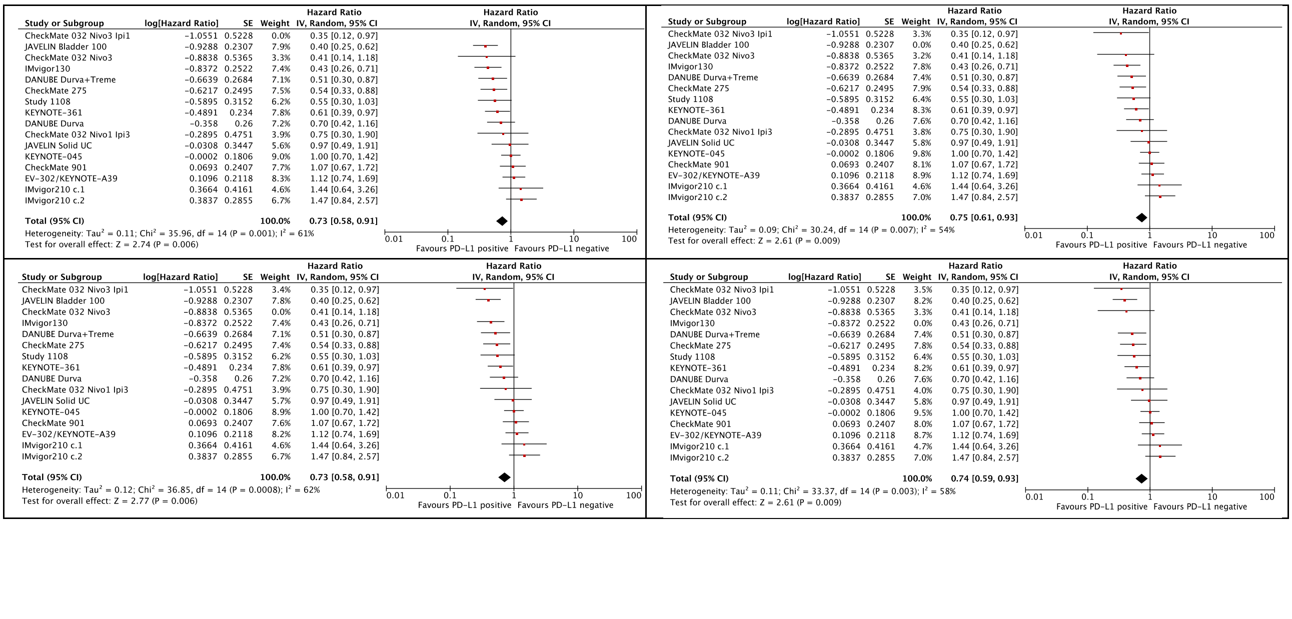

© 2024 Maiorano BA et al. *JAMA Network Open*.

Study or Subgroup	log[Hazard Ratio]	SE	Weight	Hazard Ratio IV, Random, 95% CI
CheckMate 032 Nivo3 Ipi1	−1.0551	0.5228	3.6%	0.35 [0.12, 0.97]
JAVELIN Bladder 100	−0.9288	0.2307	8.1%	0.40 [0.25, 0.62]
CheckMate 032 Nivo3	−0.8838	0.5365	3.5%	0.41 [0.14, 1.18]
IMvigor130	−0.8372	0.2522	7.7%	0.43 [0.26, 0.71]
DANUBE Durva+Treme	−0.6639	0.2684	0.0%	0.51 [0.30, 0.87]
CheckMate 275	−0.6217	0.2495	7.7%	0.54 [0.33, 0.88]
Study 1108	−0.5895	0.3152	6.4%	0.55 [0.30, 1.03]
KEYNOTE−361	−0.4891	0.234	8.1%	0.61 [0.39, 0.97]
DANUBE Durva	−0.358	0.26	7.5%	0.70 [0.42, 1.16]
CheckMate 032 Nivo1 Ipi3	−0.2895	0.4751	4.1%	0.75 [0.30, 1.90]
JAVELIN Solid UC	−0.0308	0.3447	5.9%	0.97 [0.49, 1.91]
KEYNOTE−045	−0.0002	0.1806	9.2%	1.00 [0.70, 1.42]
CheckMate 901	0.0693	0.2407	7.9%	1.07 [0.67, 1.72]
EV−302/KEYNOTE−A39	0.1096	0.2118	8.5%	1.12 [0.74, 1.69]
IMvigor210 c.1	0.3664	0.4161	4.8%	1.44 [0.64, 3.26]
IMvigor210 c.2	0.3837	0.2855	7.0%	1.47 [0.84, 2.57]
**Total (95% CI)**			**100.0%**	**0.73 [0.58, 0.92]**

Heterogeneity: $Tau^2 = 0.12$; $Chi^2 = 36.19$, df = 14 (P = 0.0010); $I^2 = 61\%$
Test for overall effect: Z = 2.65 (P = 0.008)
0.01 0.1 1 10 100 — Favours PD−L1 positive / Favours PD−L1 negative

Study or Subgroup	log[Hazard Ratio]	SE	Weight	Hazard Ratio IV, Random, 95% CI
CheckMate 032 Nivo3 Ipi1	−1.0551	0.5228	3.6%	0.35 [0.12, 0.97]
JAVELIN Bladder 100	−0.9288	0.2307	8.1%	0.40 [0.25, 0.62]
CheckMate 032 Nivo3	−0.8838	0.5365	3.5%	0.41 [0.14, 1.18]
IMvigor130	−0.8372	0.2522	7.7%	0.43 [0.26, 0.71]
DANUBE Durva+Treme	−0.6639	0.2684	7.4%	0.51 [0.30, 0.87]
CheckMate 275	−0.6217	0.2495	0.0%	0.54 [0.33, 0.88]
Study 1108	−0.5895	0.3152	6.5%	0.55 [0.30, 1.03]
KEYNOTE−361	−0.4891	0.234	8.1%	0.61 [0.39, 0.97]
DANUBE Durva	−0.358	0.26	7.5%	0.70 [0.42, 1.16]
CheckMate 032 Nivo1 Ipi3	−0.2895	0.4751	4.1%	0.75 [0.30, 1.90]
JAVELIN Solid UC	−0.0308	0.3447	5.9%	0.97 [0.49, 1.91]
KEYNOTE−045	−0.0002	0.1806	9.2%	1.00 [0.70, 1.42]
CheckMate 901	0.0693	0.2407	7.9%	1.07 [0.67, 1.72]
EV−302/KEYNOTE−A39	0.1096	0.2118	8.6%	1.12 [0.74, 1.69]
IMvigor210 c.1	0.3664	0.4161	4.8%	1.44 [0.64, 3.26]
IMvigor210 c.2	0.3837	0.2855	7.0%	1.47 [0.84, 2.57]
**Total (95% CI)**			**100.0%**	**0.73 [0.58, 0.92]**

Heterogeneity: $Tau^2 = 0.12$; $Chi^2 = 36.36$, df = 14 (P = 0.0009); $I^2 = 61\%$
Test for overall effect: Z = 2.65 (P = 0.008)
0.01 0.1 1 10 100 — Favours PD−L1 positive / Favours PD−L1 negative

Study or Subgroup	log[Hazard Ratio]	SE	Weight	Hazard Ratio IV, Random, 95% CI
CheckMate 032 Nivo3 Ipi1	−1.0551	0.5228	3.6%	0.35 [0.12, 0.97]
JAVELIN Bladder 100	−0.9288	0.2307	8.0%	0.40 [0.25, 0.62]
CheckMate 032 Nivo3	−0.8838	0.5365	3.5%	0.41 [0.14, 1.18]
IMvigor130	−0.8372	0.2522	7.6%	0.43 [0.26, 0.71]
DANUBE Durva+Treme	−0.6639	0.2684	7.3%	0.51 [0.30, 0.87]
CheckMate 275	−0.6217	0.2495	7.6%	0.54 [0.33, 0.88]
Study 1108	−0.5895	0.3152	0.0%	0.55 [0.30, 1.03]
KEYNOTE−361	−0.4891	0.234	8.0%	0.61 [0.39, 0.97]
DANUBE Durva	−0.358	0.26	7.4%	0.70 [0.42, 1.16]
CheckMate 032 Nivo1 Ipi3	−0.2895	0.4751	4.1%	0.75 [0.30, 1.90]
JAVELIN Solid UC	−0.0308	0.3447	5.9%	0.97 [0.49, 1.91]
KEYNOTE−045	−0.0002	0.1806	9.1%	1.00 [0.70, 1.42]
CheckMate 901	0.0693	0.2407	7.8%	1.07 [0.67, 1.72]
EV−302/KEYNOTE−A39	0.1096	0.2118	8.4%	1.12 [0.74, 1.69]
IMvigor210 c.1	0.3664	0.4161	4.8%	1.44 [0.64, 3.26]
IMvigor210 c.2	0.3837	0.2855	6.9%	1.47 [0.84, 2.57]
**Total (95% CI)**			**100.0%**	**0.72 [0.57, 0.91]**

Heterogeneity: $Tau^2 = 0.12$; $Chi^2 = 37.20$, df = 14 (P = 0.0007); $I^2 = 62\%$
Test for overall effect: Z = 2.71 (P = 0.007)
0.01 0.1 1 10 100 — Favours PD−L1 positive / Favours PD−L1 negative

Study or Subgroup	log[Hazard Ratio]	SE	Weight	Hazard Ratio IV, Random, 95% CI
CheckMate 032 Nivo3 Ipi1	−1.0551	0.5228	3.7%	0.35 [0.12, 0.97]
JAVELIN Bladder 100	−0.9288	0.2307	8.1%	0.40 [0.25, 0.62]
CheckMate 032 Nivo3	−0.8838	0.5365	3.6%	0.41 [0.14, 1.18]
IMvigor130	−0.8372	0.2522	7.7%	0.43 [0.26, 0.71]
DANUBE Durva+Treme	−0.6639	0.2684	7.4%	0.51 [0.30, 0.87]
CheckMate 275	−0.6217	0.2495	7.8%	0.54 [0.33, 0.88]
Study 1108	−0.5895	0.3152	6.5%	0.55 [0.30, 1.03]
KEYNOTE−361	−0.4891	0.234	0.0%	0.61 [0.39, 0.97]
DANUBE Durva	−0.358	0.26	7.5%	0.70 [0.42, 1.16]
CheckMate 032 Nivo1 Ipi3	−0.2895	0.4751	4.2%	0.75 [0.30, 1.90]
JAVELIN Solid UC	−0.0308	0.3447	6.0%	0.97 [0.49, 1.91]
KEYNOTE−045	−0.0002	0.1806	9.2%	1.00 [0.70, 1.42]
CheckMate 901	0.0693	0.2407	7.9%	1.07 [0.67, 1.72]
EV−302/KEYNOTE−A39	0.1096	0.2118	8.5%	1.12 [0.74, 1.69]
IMvigor210 c.1	0.3664	0.4161	4.9%	1.44 [0.64, 3.26]
IMvigor210 c.2	0.3837	0.2855	7.0%	1.47 [0.84, 2.57]
**Total (95% CI)**			**100.0%**	**0.72 [0.57, 0.92]**

Heterogeneity: $Tau^2 = 0.13$; $Chi^2 = 37.39$, df = 14 (P = 0.0006); $I^2 = 63\%$
Test for overall effect: Z = 2.68 (P = 0.007)
0.01 0.1 1 10 100 — Favours PD−L1 positive / Favours PD−L1 negative

Study or Subgroup	log[Hazard Ratio]	SE	Weight	Hazard Ratio IV, Random, 95% CI
CheckMate 032 Nivo3 Ipi1	−1.0551	0.5228	3.7%	0.35 [0.12, 0.97]
JAVELIN Bladder 100	−0.9288	0.2307	8.1%	0.40 [0.25, 0.62]
CheckMate 032 Nivo3	−0.8838	0.5365	3.6%	0.41 [0.14, 1.18]
IMvigor130	−0.8372	0.2522	7.7%	0.43 [0.26, 0.71]
DANUBE Durva+Treme	−0.6639	0.2684	7.3%	0.51 [0.30, 0.87]
CheckMate 275	−0.6217	0.2495	7.7%	0.54 [0.33, 0.88]
Study 1108	−0.5895	0.3152	6.5%	0.55 [0.30, 1.03]
KEYNOTE−361	−0.4891	0.234	8.0%	0.61 [0.39, 0.97]
DANUBE Durva	−0.358	0.26	0.0%	0.70 [0.42, 1.16]
CheckMate 032 Nivo1 Ipi3	−0.2895	0.4751	4.2%	0.75 [0.30, 1.90]
JAVELIN Solid UC	−0.0308	0.3447	6.0%	0.97 [0.49, 1.91]
KEYNOTE−045	−0.0002	0.1806	9.1%	1.00 [0.70, 1.42]
CheckMate 901	0.0693	0.2407	7.9%	1.07 [0.67, 1.72]
EV−302/KEYNOTE−A39	0.1096	0.2118	8.5%	1.12 [0.74, 1.69]
IMvigor210 c.1	0.3664	0.4161	4.9%	1.44 [0.64, 3.26]
IMvigor210 c.2	0.3837	0.2855	7.0%	1.47 [0.84, 2.57]
**Total (95% CI)**			**100.0%**	**0.71 [0.56, 0.91]**

Heterogeneity: $Tau^2 = 0.13$; $Chi^2 = 37.97$, df = 14 (P = 0.0005); $I^2 = 63\%$
Test for overall effect: Z = 2.77 (P = 0.006)
0.01 0.1 1 10 100 — Favours PD−L1 positive / Favours PD−L1 negative

Study or Subgroup	log[Hazard Ratio]	SE	Weight	Hazard Ratio IV, Random, 95% CI
CheckMate 032 Nivo3 Ipi1	−1.0551	0.5228	3.5%	0.35 [0.12, 0.97]
JAVELIN Bladder 100	−0.9288	0.2307	7.9%	0.40 [0.25, 0.62]
CheckMate 032 Nivo3	−0.8838	0.5365	3.4%	0.41 [0.14, 1.18]
IMvigor130	−0.8372	0.2522	7.4%	0.43 [0.26, 0.71]
DANUBE Durva+Treme	−0.6639	0.2684	7.1%	0.51 [0.30, 0.87]
CheckMate 275	−0.6217	0.2495	7.5%	0.54 [0.33, 0.88]
Study 1108	−0.5895	0.3152	6.2%	0.55 [0.30, 1.03]
KEYNOTE−361	−0.4891	0.234	7.8%	0.61 [0.39, 0.97]
DANUBE Durva	−0.358	0.26	7.3%	0.70 [0.42, 1.16]
CheckMate 032 Nivo1 Ipi3	−0.2895	0.4751	0.0%	0.75 [0.30, 1.90]
JAVELIN Solid UC	−0.0308	0.3447	5.7%	0.97 [0.49, 1.91]
KEYNOTE−045	−0.0002	0.1806	8.9%	1.00 [0.70, 1.42]
CheckMate 901	0.0693	0.2407	7.7%	1.07 [0.67, 1.72]
EV−302/KEYNOTE−A39	0.1096	0.2118	8.2%	1.12 [0.74, 1.69]
IMvigor210 c.1	0.3664	0.4161	4.7%	1.44 [0.64, 3.26]
IMvigor210 c.2	0.3837	0.2855	6.8%	1.47 [0.84, 2.57]
**Total (95% CI)**			**100.0%**	**0.71 [0.56, 0.90]**

Heterogeneity: $Tau^2 = 0.12$; $Chi^2 = 37.99$, df = 14 (P = 0.0005); $I^2 = 63\%$
Test for overall effect: Z = 2.89 (P = 0.004)
0.01 0.1 1 10 100 — Favours PD−L1 positive / Favours PD−L1 negative

Study or Subgroup	log[Hazard Ratio]	SE	Weight	Hazard Ratio IV, Random, 95% CI
CheckMate 032 Nivo3 Ipi1	−1.0551	0.5228	3.6%	0.35 [0.12, 0.97]
JAVELIN Bladder 100	−0.9288	0.2307	8.0%	0.40 [0.25, 0.62]
CheckMate 032 Nivo3	−0.8838	0.5365	3.4%	0.41 [0.14, 1.18]
IMvigor130	−0.8372	0.2522	7.6%	0.43 [0.26, 0.71]
DANUBE Durva+Treme	−0.6639	0.2684	7.2%	0.51 [0.30, 0.87]
CheckMate 275	−0.6217	0.2495	7.6%	0.54 [0.33, 0.88]
Study 1108	−0.5895	0.3152	6.3%	0.55 [0.30, 1.03]
KEYNOTE−361	−0.4891	0.234	7.9%	0.61 [0.39, 0.97]
DANUBE Durva	−0.358	0.26	7.4%	0.70 [0.42, 1.16]
CheckMate 032 Nivo1 Ipi3	−0.2895	0.4751	4.0%	0.75 [0.30, 1.90]
JAVELIN Solid UC	−0.0308	0.3447	0.0%	0.97 [0.49, 1.91]
KEYNOTE−045	−0.0002	0.1806	9.1%	1.00 [0.70, 1.42]
CheckMate 901	0.0693	0.2407	7.8%	1.07 [0.67, 1.72]
EV−302/KEYNOTE−A39	0.1096	0.2118	8.4%	1.12 [0.74, 1.69]
IMvigor210 c.1	0.3664	0.4161	4.8%	1.44 [0.64, 3.26]
IMvigor210 c.2	0.3837	0.2855	6.9%	1.47 [0.84, 2.57]
**Total (95% CI)**			**100.0%**	**0.70 [0.55, 0.88]**

Heterogeneity: $Tau^2 = 0.12$; $Chi^2 = 37.29$, df = 14 (P = 0.0007); $I^2 = 62\%$
Test for overall effect: Z = 3.00 (P = 0.003)
0.01 0.1 1 10 100 — Favours PD−L1 positive / Favours PD−L1 negative

Study or Subgroup	log[Hazard Ratio]	SE	Weight	Hazard Ratio IV, Random, 95% CI
CheckMate 032 Nivo3 Ipi1	−1.0551	0.5228	3.7%	0.35 [0.12, 0.97]
JAVELIN Bladder 100	−0.9288	0.2307	8.3%	0.40 [0.25, 0.62]
CheckMate 032 Nivo3	−0.8838	0.5365	3.5%	0.41 [0.14, 1.18]
IMvigor130	−0.8372	0.2522	7.8%	0.43 [0.26, 0.71]
DANUBE Durva+Treme	−0.6639	0.2684	7.5%	0.51 [0.30, 0.87]
CheckMate 275	−0.6217	0.2495	7.9%	0.54 [0.33, 0.88]
Study 1108	−0.5895	0.3152	6.5%	0.55 [0.30, 1.03]
KEYNOTE−361	−0.4891	0.234	8.2%	0.61 [0.39, 0.97]
DANUBE Durva	−0.358	0.26	7.7%	0.70 [0.42, 1.16]
CheckMate 032 Nivo1 Ipi3	−0.2895	0.4751	4.2%	0.75 [0.30, 1.90]
JAVELIN Solid UC	−0.0308	0.3447	6.0%	0.97 [0.49, 1.91]
KEYNOTE−045	−0.0002	0.1806	0.0%	1.00 [0.70, 1.42]
CheckMate 901	0.0693	0.2407	8.1%	1.07 [0.67, 1.72]
EV−302/KEYNOTE−A39	0.1096	0.2118	8.7%	1.12 [0.74, 1.69]
IMvigor210 c.1	0.3664	0.4161	4.9%	1.44 [0.64, 3.26]
IMvigor210 c.2	0.3837	0.2855	7.1%	1.47 [0.84, 2.57]
**Total (95% CI)**			**100.0%**	**0.69 [0.55, 0.87]**

Heterogeneity: $Tau^2 = 0.12$; $Chi^2 = 34.45$, df = 14 (P = 0.002); $I^2 = 59\%$
Test for overall effect: Z = 3.09 (P = 0.002)
0.01 0.1 1 10 100 — Favours PD−L1 positive / Favours PD−L1 negative

Study or Subgroup	log[Hazard Ratio]	SE	Weight	Hazard Ratio IV, Random, 95% CI
CheckMate 032 Nivo3 Ipi1	−1.0551	0.5228	3.6%	0.35 [0.12, 0.97]
JAVELIN Bladder 100	−0.9288	0.2307	8.2%	0.40 [0.25, 0.62]
CheckMate 032 Nivo3	−0.8838	0.5365	3.4%	0.41 [0.14, 1.18]
IMvigor130	−0.8372	0.2522	7.7%	0.43 [0.26, 0.71]
DANUBE Durva+Treme	−0.6639	0.2684	7.4%	0.51 [0.30, 0.87]
CheckMate 275	−0.6217	0.2495	7.8%	0.54 [0.33, 0.88]
Study 1108	−0.5895	0.3152	6.4%	0.55 [0.30, 1.03]
KEYNOTE−361	−0.4891	0.234	8.1%	0.61 [0.39, 0.97]
DANUBE Durva	−0.358	0.26	7.6%	0.70 [0.42, 1.16]
CheckMate 032 Nivo1 Ipi3	−0.2895	0.4751	4.1%	0.75 [0.30, 1.90]
JAVELIN Solid UC	−0.0308	0.3447	5.9%	0.97 [0.49, 1.91]
KEYNOTE−045	−0.0002	0.1806	9.3%	1.00 [0.70, 1.42]
CheckMate 901	0.0693	0.2407	0.0%	1.07 [0.67, 1.72]
EV−302/KEYNOTE−A39	0.1096	0.2118	8.6%	1.12 [0.74, 1.69]
IMvigor210 c.1	0.3664	0.4161	4.8%	1.44 [0.64, 3.26]
IMvigor210 c.2	0.3837	0.2855	7.0%	1.47 [0.84, 2.57]
**Total (95% CI)**			**100.0%**	**0.69 [0.55, 0.87]**

Heterogeneity: $Tau^2 = 0.12$; $Chi^2 = 35.22$, df = 14 (P = 0.001); $I^2 = 60\%$
Test for overall effect: Z = 3.14 (P = 0.002)
0.01 0.1 1 10 100 — Favours PD−L1 positive / Favours PD−L1 negative

Study or Subgroup	log[Hazard Ratio]	SE	Weight	Hazard Ratio IV, Random, 95% CI
CheckMate 032 Nivo3 Ipi1	−1.0551	0.5228	3.5%	0.35 [0.12, 0.97]
JAVELIN Bladder 100	−0.9288	0.2307	8.3%	0.40 [0.25, 0.62]
CheckMate 032 Nivo3	−0.8838	0.5365	3.4%	0.41 [0.14, 1.18]
IMvigor130	−0.8372	0.2522	7.8%	0.43 [0.26, 0.71]
DANUBE Durva+Treme	−0.6639	0.2684	7.4%	0.51 [0.30, 0.87]
CheckMate 275	−0.6217	0.2495	7.9%	0.54 [0.33, 0.88]
Study 1108	−0.5895	0.3152	6.5%	0.55 [0.30, 1.03]
KEYNOTE−361	−0.4891	0.234	8.2%	0.61 [0.39, 0.97]
DANUBE Durva	−0.358	0.26	7.6%	0.70 [0.42, 1.16]
CheckMate 032 Nivo1 Ipi3	−0.2895	0.4751	4.0%	0.75 [0.30, 1.90]
JAVELIN Solid UC	−0.0308	0.3447	5.9%	0.97 [0.49, 1.91]
KEYNOTE−045	−0.0002	0.1806	9.5%	1.00 [0.70, 1.42]
CheckMate 901	0.0693	0.2407	8.1%	1.07 [0.67, 1.72]
EV−302/KEYNOTE−A39	0.1096	0.2118	0.0%	1.12 [0.74, 1.69]
IMvigor210 c.1	0.3664	0.4161	4.8%	1.44 [0.64, 3.26]
IMvigor210 c.2	0.3837	0.2855	7.1%	1.47 [0.84, 2.57]
**Total (95% CI)**			**100.0%**	**0.69 [0.55, 0.86]**

Heterogeneity: $Tau^2 = 0.11$; $Chi^2 = 33.51$, df = 14 (P = 0.002); $I^2 = 58\%$
Test for overall effect: Z = 3.24 (P = 0.001)
0.01 0.1 1 10 100 — Favours PD−L1 positive / Favours PD−L1 negative

Study or Subgroup	log[Hazard Ratio]	SE	Weight	Hazard Ratio IV, Random, 95% CI
CheckMate 032 Nivo3 Ipi1	−1.0551	0.5228	3.4%	0.35 [0.12, 0.97]
JAVELIN Bladder 100	−0.9288	0.2307	8.0%	0.40 [0.25, 0.62]
CheckMate 032 Nivo3	−0.8838	0.5365	3.3%	0.41 [0.14, 1.18]
IMvigor130	−0.8372	0.2522	7.5%	0.43 [0.26, 0.71]
DANUBE Durva+Treme	−0.6639	0.2684	7.2%	0.51 [0.30, 0.87]
CheckMate 275	−0.6217	0.2495	7.6%	0.54 [0.33, 0.88]
Study 1108	−0.5895	0.3152	6.2%	0.55 [0.30, 1.03]
KEYNOTE−361	−0.4891	0.234	7.9%	0.61 [0.39, 0.97]
DANUBE Durva	−0.358	0.26	7.3%	0.70 [0.42, 1.16]
CheckMate 032 Nivo1 Ipi3	−0.2895	0.4751	3.9%	0.75 [0.30, 1.90]
JAVELIN Solid UC	−0.0308	0.3447	5.7%	0.97 [0.49, 1.91]
KEYNOTE−045	−0.0002	0.1806	9.1%	1.00 [0.70, 1.42]
CheckMate 901	0.0693	0.2407	7.8%	1.07 [0.67, 1.72]
EV−302/KEYNOTE−A39	0.1096	0.2118	8.4%	1.12 [0.74, 1.69]
IMvigor210 c.1	0.3664	0.4161	0.0%	1.44 [0.64, 3.26]
IMvigor210 c.2	0.3837	0.2855	6.8%	1.47 [0.84, 2.57]
**Total (95% CI)**			**100.0%**	**0.69 [0.55, 0.86]**

Heterogeneity: $Tau^2 = 0.11$; $Chi^2 = 35.24$, df = 14 (P = 0.001); $I^2 = 60\%$
Test for overall effect: Z = 3.24 (P = 0.001)
0.01 0.1 1 10 100 — Favours PD−L1 positive / Favours PD−L1 negative

Study or Subgroup	log[Hazard Ratio]	SE	Weight	Hazard Ratio IV, Random, 95% CI
CheckMate 032 Nivo3 Ipi1	−1.0551	0.5228	3.3%	0.35 [0.12, 0.97]
JAVELIN Bladder 100	−0.9288	0.2307	8.3%	0.40 [0.25, 0.62]
CheckMate 032 Nivo3	−0.8838	0.5365	3.2%	0.41 [0.14, 1.18]
IMvigor130	−0.8372	0.2522	7.7%	0.43 [0.26, 0.71]
DANUBE Durva+Treme	−0.6639	0.2684	7.3%	0.51 [0.30, 0.87]
CheckMate 275	−0.6217	0.2495	7.8%	0.54 [0.33, 0.88]
Study 1108	−0.5895	0.3152	6.3%	0.55 [0.30, 1.03]
KEYNOTE−361	−0.4891	0.234	8.2%	0.61 [0.39, 0.97]
DANUBE Durva	−0.358	0.26	7.5%	0.70 [0.42, 1.16]
CheckMate 032 Nivo1 Ipi3	−0.2895	0.4751	3.8%	0.75 [0.30, 1.90]
JAVELIN Solid UC	−0.0308	0.3447	5.7%	0.97 [0.49, 1.91]
KEYNOTE−045	−0.0002	0.1806	9.6%	1.00 [0.70, 1.42]
CheckMate 901	0.0693	0.2407	8.0%	1.07 [0.67, 1.72]
EV−302/KEYNOTE−A39	0.1096	0.2118	8.7%	1.12 [0.74, 1.69]
IMvigor210 c.1	0.3664	0.4161	4.6%	1.44 [0.64, 3.26]
IMvigor210 c.2	0.3837	0.2855	0.0%	1.47 [0.84, 2.57]
**Total (95% CI)**			**100.0%**	**0.68 [0.55, 0.84]**

Heterogeneity: $Tau^2 = 0.09$; $Chi^2 = 31.64$, df = 14 (P = 0.005); $I^2 = 56\%$
Test for overall effect: Z = 3.50 (P = 0.0005)
0.01 0.1 1 10 100 — Favours PD−L1 positive / Favours PD−L1 negative

© 2024 Maiorano BA et al. *JAMA Network Open*.

C

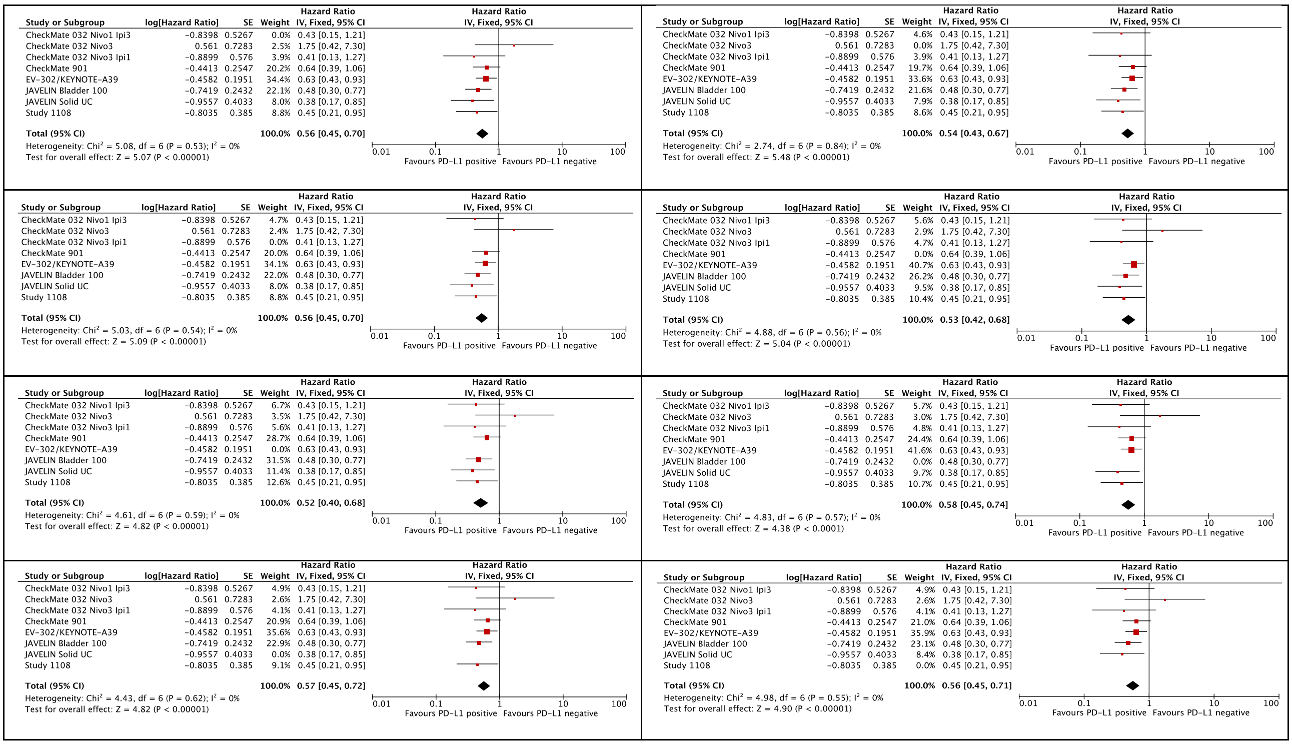

eFigure 4. Sensitivity Analysis for (A) ORR, (B) OS, and (C) PFS by Removing One Study at a Time

© 2024 Maiorano BA et al. *JAMA Network Open*.
