# Supplementary material for: Significance of PD-L1 in Metastatic Urothelial Carcinoma Treated With Immune Checkpoint Inhibitors: A Systematic Review and Meta-Analysis
Source: JAMA Netw Open. 2024 Mar 6;7(3):e241215. doi: 10.1001/jamanetworkopen.2024.1215 (PMC10918499; doi:10.1001/jamanetworkopen.2024.1215)
Supplement: Supplement 2. — Nonauthor Collaborators [file jamanetwopen-e241215-s002.pdf]

Supplemental Online Content: Nonauthor Collaborators
*First name, last name, and suffix (if applicable) are required and will appear in PubMed.

*Group Name(s): MeetUro Group							
***First Name and Middle Initial(s)**	***Last Name**	***Suffix (eg, Jr, III)**	Academic Degrees	Institution	Location (city, state/province, country)	Role or Contribution, eg, chair, principal investigator	Group (if more than 1 Group listed in the byline) and/or Subgroup (eg, Steering Committee)
Caterina	Accettura	/					
Michele	Aieta						
Martina	Alberti						
Marta	Aliprandi						
Amelia	Altavilla						
Lorenzo	Antonuzzo						
Adele	Artemi						
Dario	Arundine						
Serena	Astore						
Laura	Attademo						
Francesco	Atzori						
Gaetano	Aurilio						
Amalia	Azzariti						
Giulia	Baciarello						
Susanne	Baier						
Valentina	Baldazzi						
Giuseppe L	Banna						
Carmen	Barile						
Salvina	Barra						
Chiara	Barraco						
Debora	Basile						
Maria	Bassanelli						
Umberto	Basso						
Matteo	Bauckneht						
Alessandra	Bearz						
Salvatore R	Bellia						
Benedetta	Benedetti						
Rossana	Berardi						
Marco	Bergamini						

Supplemental Online Content: Nonauthor Collaborators
*First name, last name, and suffix (if applicable) are required and will appear in PubMed.

***First Name and Middle Initial(s)**	***Last Name**	***Suffix (eg, Jr, III)**	Academic Degrees	Institution	Location (city, state/province, country)	Role or Contribution, eg, chair, principal investigator	Group (if more than 1 Group listed in the byline) and/or Subgroup (eg, Steering Committee)
Melissa	Bersanelli						
Maria	Bertoni						
Emanuela	Bianchi						
Claudia	Biasini						
Livia	Bidin						
Davide	Bimbatti						
Sara	Bleve						
Francesco	Boccardo						
Elena	Bolzacchini						
Adele	Bonato						
Lucia	Bonomi						
Sebastiano	Bordonaro						
Marco	Borghesi						
Roberto	Bortolus						
Lucia	Bortot						
Davide	Bosso						
Achille	Bottiglieri						
Giovanni	Bozza						
Marco	Bregni						
Nicole	Brighi						
Enrico	Bronte						
Alessio	Bruni						
Michele	Bruno						
Martina	Buffoni						
Luciana	Buonerba						
Carlo	Buonerba						
Sebastiano	Buti						
Consuelo	Buttigliero						
Jessica	Cadau						
Orazio	Caffo						
Fabio	Calabrò						

Supplemental Online Content: Nonauthor Collaborators
*First name, last name, and suffix (if applicable) are required and will appear in PubMed.

***First Name and Middle Initial(s)**	***Last Name**	***Suffix (eg, Jr, III)**	Academic Degrees	Institution	Location (city, state/province, country)	Role or Contribution, eg, chair, principal investigator	Group (if more than 1 Group listed in the byline) and/or Subgroup (eg, Steering Committee)
Nicola	Calvani						
Davide	Campobasso						
Samanta	Capacci						
Umberto	Capitanio						
Salvatore R	Caponnetto						
Claudia	Carella						
Paolo	Carlini						
Francesco	Carrozza						
Giacomo	Cartenì						
Davide	Caruso						
Chiara	Casadei						
Fabio	Catalano						
Martina	Catalano						
Carlo	Cattrini						
Nicolò	Cavasin						
Alessia	Cavo						
Luigi G	Cecchi						
Sabrina C	Cecere						
Linda	Cerbone						
Giovanni L	Ceresoli						
Rita	Chiari						
Silvia	Chiellino						
Vincenzo E	Chiuri						
Stefano	Ciccarelli						
Giuseppe L	Cicero						
Marika	Cinausero						
Fabrizio	Citarella						
Melanie	Claps						
Emilia	Cocorocchio						
Vincenza	Conteduca						
Elisabetta	Coppola						

Supplemental Online Content: Nonauthor Collaborators
*First name, last name, and suffix (if applicable) are required and will appear in PubMed.

***First Name and Middle Initial(s)**	***Last Name**	***Suffix (eg, Jr, III)**	Academic Degrees	Institution	Location (city, state/province, country)	Role or Contribution, eg, chair, principal investigator	Group (if more than 1 Group listed in the byline) and/or Subgroup (eg, Steering Committee)
Nadia	Cordua						
Maria	Cossu Rocca						
Giulia	Courthod						
Malvina	Cremante						
Daniela	Cullurà						
Maria C	Cursano						
Nicola	D'Ostilio						
Alberto	Dalla Volta						
Alessandra	Damassi						
Bruno	Daniele						
Ugo FF	De Giorgi						
Mariagrazia	De Lisa						
Delia	De Lisi						
Simona	De Summa						
Rocco	De Vivo						
Silvia A	Debonis						
Marzia	Del Re						
Ilaria	Depetris						
Mattia A	Di Civita						
Fabrizio	Di Costanzo						
Simona	Di Francesco						
Stefania	Di Girolamo						
Giuseppe	Di Lorenzo						
Massimo	Di Maio						
Marilena	Di Napoli						
Roberta	Di Rocco						
Piergiorgio	Di Tullio						
Angelo	Dinota						
Michele	Dionese						
Donatella	Donati						
Laura	Doni						

Supplemental Online Content: Nonauthor Collaborators
*First name, last name, and suffix (if applicable) are required and will appear in PubMed.

***First Name and Middle Initial(s)**	***Last Name**	***Suffix (eg, Jr, III)**	Academic Degrees	Institution	Location (city, state/province, country)	Role or Contribution, eg, chair, principal investigator	Group (if more than 1 Group listed in the byline) and/or Subgroup (eg, Steering Committee)
Antonio	Doronzo						
Arianna	Dri						
Paola	Ermacora						
Laura	Evangelista						
Martina	Fanelli						
Elena	Farè						
Azzurra	Farnesi						
Alberto	Farolfi						
Antonio	Febbraro						
Palma	Fedele						
Piera	Federico						
Gilberto	Filaci						
Roberto	Filippi						
Alessia	Filograna						
Simone	Foderaro						
Antonella	Fontana						
Luigi	Formisano						
Giuseppe	Fornarini						
Edoardo	Francini						
Ciro	Franzese						
Lucia	Fratino						
Daniele	Galanti						
Luca	Galli						
Teresa	Gamba						
Elisabetta	Gambale						
Silvio K	Garattini						
Elisabetta	Garibaldi						
Donatello	Gasparro						
Anna L	Gentile						
Domenico	Germano						
Angela	Gernone						

Supplemental Online Content: Nonauthor Collaborators
*First name, last name, and suffix (if applicable) are required and will appear in PubMed.

***First Name and Middle Initial(s)**	***Last Name**	***Suffix (eg, Jr, III)**	Academic Degrees	Institution	Location (city, state/province, country)	Role or Contribution, eg, chair, principal investigator	Group (if more than 1 Group listed in the byline) and/or Subgroup (eg, Steering Committee)
Laura	Ghilardi						
Patrizia	Giannatempo						
Caterina	Gianni						
Emilia	Gianotti						
Roberta	Giorgione						
Giulia C	Giudice						
Emilio F	Giunta						
Cesare	Gridelli						
Francesco	Grillone						
Valentina	Guadalupi						
Simona	Gualtieri						
Alessia S	Guarneri						
Priscilla	Guglielmo						
Alketa	Hamzaj						
Afete	Hamzaj						
Maria L	Iaia						
Debora	Ierinò						
Lorena	Incorvaia						
Concetta	Ingenito						
Nicola	Inzerilli						
Salim	Jubran						
Stefania	Kinspergher						
Antonella	La Camera						
Francesca	La Russa						
Eleonora	Lai						
Federica	Lancia						
Matteo	Landriscina						
Francesco	Lanfranchi						
Fiorenza	Latteri						
Edoardo	Lenci						
Helga MA	Lipari						

Supplemental Online Content: Nonauthor Collaborators
*First name, last name, and suffix (if applicable) are required and will appear in PubMed.

***First Name and Middle Initial(s)**	***Last Name**	***Suffix (eg, Jr, III)**	Academic Degrees	Institution	Location (city, state/province, country)	Role or Contribution, eg, chair, principal investigator	Group (if more than 1 Group listed in the byline) and/or Subgroup (eg, Steering Committee)
Migual A	Llaja Obispo						
Monica	Lo Vecchio						
Valentina N	Lombardo						
Laura	Lombardo						
Andrea	Luciani						
Marianna	Macerelli						
Michele	Maffezzoli						
Francesca	Maines						
Brigida A	Maiorano						
Andrea	Malgeri						
Daniele	Marinelli						
Marco	Maruzzo						
Cristina	Masini						
Daiana	Massi						
Alessandro	Mastrorosa						
Alvise	Mattana						
Manlio	Mencoboni						
Alessia	Mennitto						
Chiara	Mercinelli						
Carlo	Messina						
Marco	Messina						
Fortuna	Migliaccio						
Marco	Migliari						
Benedetta	Montagna						
Daniela	Montanari						
Anna M	Morelli						
Franco	Morelli						
Stefano	Moroso						
Alessandra	Mosca						
Claudia	Mucciarini						
Fernando M	Hernandez						

Supplemental Online Content: Nonauthor Collaborators
*First name, last name, and suffix (if applicable) are required and will appear in PubMed.

***First Name and Middle Initial(s)**	***Last Name**	***Suffix (eg, Jr, III)**	Academic Degrees	Institution	Location (city, state/province, country)	Role or Contribution, eg, chair, principal investigator	Group (if more than 1 Group listed in the byline) and/or Subgroup (eg, Steering Committee)
Viviana	Murgia						
Veronica	Murianni						
Maura	Murru						
Andrea	Muto						
Emanuele	Naglieri						
Cecilia	Nasso						
Maurizio	Nicodemo						
Franco	Nolè						
Valentina	Orlando						
Cinzia	Ortega						
Elisabetta	Paccagnella						
Roberto	Pacelli						
Antonio	Palazzo						
Erica	Palesandro						
Francesco	Pantano						
Federico	Paolieri						
Maria N	Pappagallo						
Laura	Pappalardo						
Antonella	Pasqualini						
Rodolfo	Passalacqua						
Anna	Passarelli						
Giovanna	Pecoraro						
Paolo	Pedrazzoli						
Giulia M	Pelin						
Antonio	Pellino						
Alessio	Pepe						
Marco	Perna						
Matteo	Perrino						
Francesco	Perrone						
Bruno	Perrucci						
Mara	Persano						

Supplemental Online Content: Nonauthor Collaborators
*First name, last name, and suffix (if applicable) are required and will appear in PubMed.

***First Name and Middle Initial(s)**	***Last Name**	***Suffix (eg, Jr, III)**	Academic Degrees	Institution	Location (city, state/province, country)	Role or Contribution, eg, chair, principal investigator	Group (if more than 1 Group listed in the byline) and/or Subgroup (eg, Steering Committee)
Alessandra	Piancastelli						
Maria	Picciotto						
Francesco	Pierantoni						
Sandro	Pignata						
Brunella	Pilato						
Margherita	Piras						
Annagrazia	Pireddu						
Chiara	Pisano						
Carmela	Pisano						
Annamaria	Piscazzi						
Salvatore R	Pisconti						
Marco	Pisino						
Alessandra	Pitrè						
Maria L	Poeta						
Luca	Pompella						
Giulia	Poti						
Veronica	Prati						
Giuseppe	Procopio						
Livio	Puglia						
Giorgia	Razzini						
Sara E	Rebuzzi						
Federica	Recine						
Pasquale	Rescigno						
Dario	Ribera						
Riccardo	Ricotta						
Alessio	Rizzo						
Mimma	Rizzo						
Michela	Roberto						
Luisa	Rollo						
Sabrina	Rossetti						
Virginia	Rossi						

Supplemental Online Content: Nonauthor Collaborators
*First name, last name, and suffix (if applicable) are required and will appear in PubMed.

***First Name and Middle Initial(s)**	***Last Name**	***Suffix (eg, Jr, III)**	Academic Degrees	Institution	Location (city, state/province, country)	Role or Contribution, eg, chair, principal investigator	Group (if more than 1 Group listed in the byline) and/or Subgroup (eg, Steering Committee)
Ernesto	Rossi						
Lorena	Rossi						
Giovanni	Rosti						
Giandomenico	Roviello						
Elvio G	Russi						
Nello	Salesi						
Alessia	Salfi						
Enrico	Sammarco						
Francesca	Sanguedolce						
Camilla	Sansi						
Fiorenza	Santamaria						
Daniele	Santini						
Matteo	Santoni						
Donata	Sartori						
Camilla	Sartragno						
Teodoro	Sava						
Andrea	Sbrana						
Sarah	Scagliarini						
Stefania	Scala						
Giuseppa	Scandurra						
Daniela	Scattolin						
Giuseppe	Schepisi						
Clorinda	Schettino						
Giovanni	Schinzari						
Simona	Secondino						
Pierangela	Sepe						
Riccardo	Serra						
Alessio	Signori						
Giustino	Silvestro						
Zuzana	Sirotovà						
Mariella	Sorarù						

Supplemental Online Content: Nonauthor Collaborators
*First name, last name, and suffix (if applicable) are required and will appear in PubMed.

***First Name and Middle Initial(s)**	***Last Name**	***Suffix (eg, Jr, III)**	Academic Degrees	Institution	Location (city, state/province, country)	Role or Contribution, eg, chair, principal investigator	Group (if more than 1 Group listed in the byline) and/or Subgroup (eg, Steering Committee)
Hector	Soto Parra						
Gian P	Spinelli						
Marco	Stellato						
Luciano	Stumbo						
Pierosandro	Tagliaferri						
Rosa	Tambaro						
Stefano	Tamberi						
Irene	Testi						
Ilaria	Toma						
Mirko	Tomasino						
Stefania	Tommasi						
Vincenzo	Tortora						
Anna	Tortorella						
ilaria	Toscani						
Paolo	Tralongo						
Renza	Triolo						
Marcello	Tucci						
Susanna	Urban						
Luca	Urso						
Anna A	Valsecchi						
Antonello	Veccia						
Chiara	Vela						
Jole	Ventriglia						
Antonio	Verde						
Elenza	Verzoni						
Francesca	Vignani						
Maria G	Vitale						
Isabella	Vittimberga						
Francesca	Zacchi						
Giulia	Zago						
Vittorina	Zagonel						

Supplemental Online Content: Nonauthor Collaborators
*First name, last name, and suffix (if applicable) are required and will appear in PubMed.

***First Name and Middle Initial(s)**	***Last Name**	***Suffix (eg, Jr, III)**	Academic Degrees	Institution	Location (city, state/province, country)	Role or Contribution, eg, chair, principal investigator	Group (if more than 1 Group listed in the byline) and/or Subgroup (eg, Steering Committee)
Elisa	Zanardi						
Diego	Zara						
Annalisa	Zeppellini						
Antonia	Zonno						
Paola A	Zucali						
Lucrezia	Zumstein						
