# Supplementary material for: Significance of PD-L1 in Metastatic Urothelial Carcinoma Treated With Immune Checkpoint Inhibitors: A Systematic Review and Meta-Analysis
Source: JAMA Netw Open. 2024 Mar 6;7(3):e241215. doi: 10.1001/jamanetworkopen.2024.1215 (PMC10918499; doi:10.1001/jamanetworkopen.2024.1215)
Supplement: Supplement 3. — Data Sharing Statement [file jamanetwopen-e241215-s003.pdf]

# Data Sharing Statement

Maiorano. Significance of PD-L1 in Metastatic Urothelial Carcinoma Treated With Immune Checkpoint Inhibitors. *JAMA Netw Open*. Published March 06, 2024. doi:10.1001/jamanetworkopen.2024.1215

**Data**
**Data available:** No

**Additional Information**
**Explanation for why data not available:** We pooled data that are already available from the clinical trials that we cited in the manuscript
